# Structural Characterisation and Chemical Stability of Commercial Fibrous Carbons in Molten Lithium Salts

**DOI:** 10.3390/ma12244232

**Published:** 2019-12-17

**Authors:** Blagoj Karakashov, Vanessa Fierro, Sandrine Mathieu, Philippe Gadonneix, Ghouti Medjahdi, Alain Celzard

**Affiliations:** 1 Institut Jean Lamour, Université de Lorraine, CNRS, IJL, F-88000 Epinal, France; blagoj.karakashov@univ-lorraine.fr (B.K.); vanessa.fierro@univ-lorraine.fr (V.F.); philippe.gadonneix@univ-lorraine.fr (P.G.); 2 Institut Jean Lamour, Université de Lorraine, CNRS, IJL, F-54000 Nancy, France; sandrine.mathieu@univ-lorraine.fr (S.M.); ghouti.medjahdi@univ-lorraine.fr (G.M.)

**Keywords:** carbon fibres, carbon felts, characterisation, chemical stability, high-temperature, lithium salts, peritectic compounds, thermal energy storage

## Abstract

The growing trend towards sustainable energy production, while intermittent, can meet all the criteria of energy demand through the use and development of high-performance thermal energy storage (TES). In this context, high-temperature hybrid TES systems, based upon the combination of fibrous carbon hosts and peritectic phase change materials (PCMs), are seen as promising solutions. One of the main conditions for the operational viability of hybrid TES is the chemical inertness between the components of the system. Thus, the chemical stability and compatibility of several commercial carbon felts (CFs) and molten lithium salts are discussed in the present study. Commercial CFs were characterised by elemental analysis, X-ray diffraction (XRD) and Raman spectroscopy before being tested in molten lithium salts: LiOH, LiBr, and the LiOH/LiBr peritectic mixture defined as our PCM of interest. The chemical stability was evaluated by gravimetry, gas adsorption and scanning electron microscopy (SEM). Among the studied CFs, the materials with the highest carbon purity and the most graphitic structure showed improved stability in contact with molten lithium salts, even under the most severe test conditions (750 °C). The application of the Arrhenius law allowed calculating the activation energy (in the range of 116 to 165 kJ mol^−1^), and estimating the potential stability of CFs at actual application temperatures. These results confirmed the applicability of CFs as porous hosts for stabilising peritectic PCMs based on molten lithium salts.

## 1. Introduction

Population growth and the economic and industrial development of many countries around the world require and support the search for constant and stable sources of energy [1]. More importantly, global concerns about climate change or pollution make it crucial to develop new technologies and sustainable materials that improve yields, especially for storing renewable energy in its various forms. In recent decades, important scientific work has been done to meet the pressing demand for alternative technologies for the collection and storage of sustainable energies, such as solar energy [2,3,4]. Thermal Energy Storage (TES) is therefore considered as one of the main solutions to meet storage expectations [5]. The latter system should play an important role in the development of clean energy storage technologies, in the improvement of global energy management and in the incorporation of intermittent solar energy, or other sources of thermal energy, thus releasing a large excess of energy that is even more sustainable [6,7].

To date, there are three types of TES systems, classified as sensible heat, latent heat and thermal chemical storage systems, or a combination of these [8]. In TES, latent heat, and more particularly phase-change materials (PCMs), play a key role in the implementation of this type of energy storage [9]. The storage via PCMs takes advantage of endothermic and exothermic isothermal phase-change processes (e.g., solid to liquid and vice-versa), exploiting increased energy densities involved at much higher temperatures than in sensible heat storage systems [10]. Thus, recent scientific advances make PCMs extremely attractive and effective for large-scale energy storage, with a small environmental footprint and a competitive price compared to other solutions [11].

Peritectic compounds (PCs) have been recently recognised as a different subcategory of PCMs, because they store energy, not only by melting/solidification, but also by a liquid/solid chemical reaction [11,12]. Indeed, PCs can be used as a one-pot storage solution, in which the chemical reaction is driven by temperature and carried out at atmospheric pressure, both in charge and discharge cycles, considered advantageous compared to gas–solid thermal chemical reaction storage systems [12]. The breakthrough of these promising PCs opens a new route for the development of novel ultra-compact systems, through the joint use of sensible, latent and thermo-chemical reaction, with high potential to provide economically viable TES [12].

However, previous studies have shown that the best performance of such PCMs and the improvement of their stability and efficiency can only be achieved by using host materials to confine them [13]. Thus, the use of carbonaceous materials, in the form of carbon fibres, carbon nanotubes, expanded graphite, graphene, alone or mixed together or with other materials, for the preparation of hybrid materials, is one of the most explored methods for improving PCMs [14]. Many properties of carbon-based materials, such as high chemical stability, low density and lower cost compared to different metals and ceramics, are indeed favourable for their use as supports of PCMs [15]. Furthermore, the use of fibrous carbons as porous hosts for PCMs, such as non-woven carbon felts (CFs), is considered a suitable solution in terms of cost, technological readiness, ability to be modified, and properties to achieve the requested TES performance [16]. 

The chemical inertness between the carbon host and the storage material is a critical factor in maintaining complete and reversible peritectic reactions, i.e., in the long-term, the effective operation of PC systems. Previously, the chemical stability of carbon fibres was considered a proven fact for their use as hosts of low-temperature organic PCMs [17,18,19,20,21,22,23,24]. However, future use of CFs in hybrid applications requires information on the compatibility and stability of composites under high-temperature working conditions and in the presence of anhydrous inorganic PCs. Some of the recently reported PCs [12] and other PCMs could potentially be corrosive in certain circumstances, depending in particular on the increase in the temperature of use, the presence of impurities (moisture, oxygen), and the occurrence of chemical reactions with carbon host materials [10,25,26]. Nowadays, the chemical compatibility of TES supports made of ceramics, inert asbestos or metals with molten inorganic PCMs, such as hydroxides, fluorides, carbonates, sulphates, chlorides, or their mixtures, is extensively studied, and the results have reported the technical challenges for developing long-lasting TES systems [27,28,29]. On the other hand, the chemical stability of carbon host candidates for high-temperature TES, via PCMs, and more specifically, via PCs, is not well examined, and therefore in-depth studies are required to test their chemical stability.

Consequently, the main purpose of this paper is the study of the materials’ properties in terms of selection criteria for chemically-stable fibrous carbons, as well as the search for the probable reasons for the occurrence of unwanted reactions between the investigated carbon host and the PC of interest here. This paper therefore proposes the necessary examination of the elemental, structural and textural properties of CFs and their constituents, i.e., the carbon fibres of ex-polyacrylonitrile (PAN) or ex-Rayon origins, and studies of their high-temperature chemical stability in molten lithium salts, LiOH and LiBr, and their peritectic phase, Li_4_(OH)_3_Br. Research in the literature has revealed that there is no similar systematic study on the effect of carbon fibre properties on their chemical stability in contact with these salts in the molten state. Li_4_(OH)_3_Br is a unique inorganic anhydrous binary system of stoichiometric composition, recently considered as a promising peritectic PCM intermediate for storing the heat produced by concentrated solar power plants or other thermal energy sources [30]. The last part of the study deals with the kinetics of the chemical reactions, using the Arrhenius law, thus considering the influence of temperature upon the chemical reaction rate. Fitting the results from CFs’ chemical reactivity data in molten LiOH, the main purpose was to obtain information on the apparent activation energy of the dominant chemical reaction, thus making it possible to estimate the burn-off of the carbon in the temperature range of the application.

## 2. Materials and Methods

### 2.1. Materials

In the present work, 12 commercial carbon felts (CFs), received from five different suppliers, were sorted into groups for an easier evaluation of their characteristics (see Table 1). In the context of this work focusing on particular CFs, Karakashov et al. (2019) [31] recalled how they are produced, and how the origin of the materials and their manufacturing history can affect their final structural properties, and their physical properties in general.

A preliminary de-sizing [32,33] step was necessary to ensure the purity (in terms of carbon content) of the commercial CFs, thus avoiding the risk of additional chemical contamination and unwanted reactions with molten inorganic salts. Therefore, slow pyrolysis (heating rate of 3 °C min^−1^ up to 900 °C followed by a dwell time of 2 h, under a flow of pure N_2_) has always been performed after receiving the CFs and before doing any further materials’ analysis. The set point of 900 °C was chosen, since the CFs were already industrially heat-treated at higher temperatures (1000–2400 °C), and thus was considered high enough for the carbonisation of any kind of polymer coating.

### 2.2. Chemical Reactivity Tests

Prior to the chemical reactivity tests, the inorganic powdered salts LiOH (anhydrous, ≥ 98%, Alfa Aesar, Kandel, Germany, melting temperature 462 °C) and LiBr (anhydrous, ≥ 99.8%, Alfa Aesar, city, country, melting temperature 550 °C) were always handled inside a glove bag (Glas-Col TM 108D X-27-27H, Terre Haute, IN, USA) under inert atmosphere because of the hygroscopic behaviour of these salts, and heated up to 120 °C to eliminate any unwanted water content.

To test the chemical stability of CFs in the molten peritectic compound (PC), an additional synthesis step was carried out by mixing LiOH and LiBr at their stoichiometric weight ratios (45 wt % LiOH/55 wt % LiBr), then synthesising the PC according to the protocol of Achchaq et al. (2019) [30]. This additional synthesis allows the formation of Li_4_(OH)_3_Br at its very precise stoichiometric composition, which is required for testing its effect on the investigated CFs. 

Indeed, if a simple physical melting had been performed prior to the chemical reactivity studies, different experimental results could possibly be obtained because of the different melting temperatures of LiOH and LiBr.

**Figure 1:**
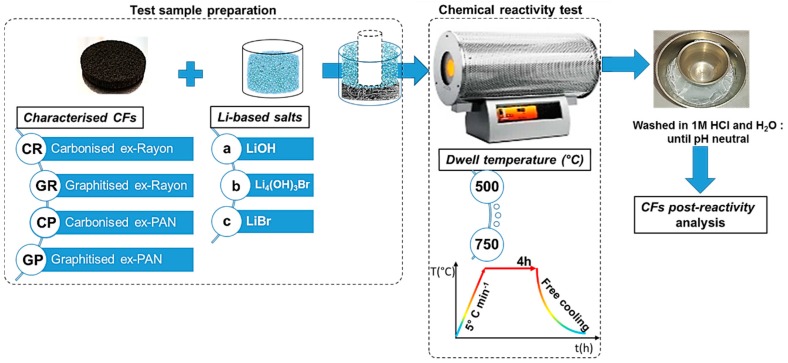
Chemical reactivity test protocol of carbon felts (CFs) in molten Li-based salts.

With regard to the chemical reactivity test (shown schematically in Figure 1), the CFs with predefined dimensions were first placed in a nickel crucible (50 mL, VWR), then covered with calculated and fixed weight ratios of LiOH/CF (40:1), LiBr/CF (80:1) or Li_4_(OH)_3_Br/CF(40:1). Separate tests were performed with LiOH and LiBr to observe the effect of the different natures of these inorganic salts [34]. By doing so, a conclusion can be drawn for the final chemical stability of the CFs in molten Li_4_(OH)_3_Br. The other important experimental variable was the heat-treatment temperature, between 500 and 750 °C. The available information presented in the literature [35] is therefore used to establish the limits of the test and the occurrence of chemical reactions, significantly controlled by the temperature, thus clearly distinguishing the different CFs. In addition, to ensure complete impregnation, an additional smaller nickel crucible (10 mL, VWR) was placed on the lightweight CF to keep it still at the bottom and in contact with the molten LiOH, LiBr or Li_4_(OH)_3_Br. In this context, Achchaq et al. (2018) [36] used a simple infiltration protocol to confirm the complete infiltration process of the nonwoven CFs with the molten PC, and the corresponding process was also successfully performed. Then, the prepared test samples were transferred to a horizontal stainless steel tubular reactor (Carbolite Gero furnace, Neuhausen, Germany) constantly under an N_2_ flow rate of 100 mL min^−1^. The reactivity tests consisted of heating the sample from room temperature to different plateau temperatures (500–750 °C), with a heating rate of 5 °C min^−1^ and a dwell time of 4h, before free cooling to ambient temperature. The solid salt block recovered with the CF immobilised therein was dissolved in a beaker filled with 2 L deionized water at 80 °C overnight. The liberated CF was transferred into a funnel with installed filter paper (Carl Roth GmbH+Co., Rotilabo^®^—round filter, type 114A, ∅ 70 mm, Karlsruhe, Germany) and washed with 1 mol L^−1^ HCl to neutral pH, and then dried at 105 °C overnight.

The examined CFs were labelled according to the scheme presented in Figure 1. Whether carbonised (i.e., manufactured at a final production temperature of less than 1600 °C) or graphitised (i.e., manufactured at a final production temperature above 2000 °C), the first part of the CF code consisted of a capital letter G or C, respectively. The latter was followed by another capital letter, relating to the carbon fibre precursor (R for Rayon and P for polyacrylonitrile (PAN)), but also the number linking the sample with the commercial supplier code (see Table 1). After performing the chemical reactivity experiments, the presented code labels were followed by a low-case letter relating to the molten salts tested as chemical reagents (a for LiOH, b for Li_4_(OH)_3_Br, or c for LiBr), ending with the temperature used. Thus, for instance, CR1a500 refers to the carbonised CF from the Rayon precursor, obtained from CeraMaterials, after a reactivity test in molten LiOH carried out at 500 °C.

### 2.3. Characterisation

#### 2.3.1. Elemental Analysis

The ultimate composition of CFs was determined using a Vario EL Cube (Elementar, Langenselbold, Germany) elemental analyser. At least two samples of each CF were analysed to check the repeatability of the results. The weight fractions of C, H, N and S were determined from the gases obtained by burning a piece of sample in an excess of dioxygen at a temperature of about 1700 °C, using as the combustion catalyst a tin foil in which the samples were wrapped. Gaseous CO_2_ and H_2_O were directly obtained, whereas N_2_ and SO_2_ were produced after reduction of NO_x_ and SO_x_ on hot copper chips. These four gases were separated and quantified by a chromatographic column. As the amount of oxygen present in the sample could not be determined in the same run, additional samples were burnt, and the resultant CO_2_ and H_2_O were reduced into CO + H_2_, and the quantification of CO led to the oxygen fraction in the materials. The same analysis was also used to observe changes in the elemental content of de-sized CFs with respect to as-received (pristine) CFs.

#### 2.3.2. X-ray Diffraction (XRD)

High-resolution X-ray diffraction (XRD) was used to get the CFs’ diffraction patterns, and was performed with a Bruker D8 Advance diffractometer (Billerica, MA, USA), at 50 kV and 45 mA, using a Mo anode (λ = 0.7093 Å) and a Lynxeye detector. The diffraction patterns were recorded in the 2*θ* range between 8 and 45° (with a scan step of 0.009°) at room temperature.

From the XRD patterns of the examined CFs’ materials, parameters such as interlayer spacing (d_002_) and crystallite size perpendicular to the carbon layers (L_c_) were calculated from the position and the width of the (002) band, respectively. The Bragg equation [37]:

(1) $$d_{002}=\frac{\lambda }{2\sin\theta }$$

where λ is the wavelength of the used radiation (λ = 0.7093 Å) and *θ* is the Bragg angle of the (002) reflection, was thus used to obtain the value of d_002_, whereas L_c_ was calculated from the conventional Scherrer equation [38]:

(2) $$L_{c}=\frac{K\lambda }{\mathrm{Bcos}\theta }$$

where K is a dimensionless shape constant with a present value of 0.9, and B is the full width at half maximum (FWHM) of the (002) reflection.

#### 2.3.3. Raman Spectroscopy

The Raman spectra of selected carbonised and graphitised CFs were studied by performing Raman scattering with a Horiba Scientific XploRa Raman spectrometer (Kyoto, Japan), carried out on samples without additional preparation. The investigation was performed for both the outer surface and cross-section of carbon fibres (see Figure S1). The dispersion of the Raman-scattered light was done by a 1200 lines per mm holographic grating and detected by a CCD camera. A sample of silicon (521 ± 2 cm^−1^) was used as a reference material. The Raman spectra were recorded with a laser at a wavelength of 532 nm, circularly polarised and filtered at 10% of its nominal power. Such filtration led to an incident power too low to overheat and damage the samples, and thus to influence the results. Once the measurement conditions were established, each sample spectrum was obtained by accumulating two spectra recorded in the range of 800 to 3500 cm^−1^. The deconvolution of the bands was carried out with Horiba’s LabSpec 6 software (Horiba France SAS, Longjumeau, France).

Typical Raman spectra of partially-graphitised or graphitised carbon materials comprise two main parts. The first order comprises D_1_ band (around 1350 cm^−1^), G band (around 1600 cm^−1^) and sometimes D_2_ band (1620 cm^−1^), and the second-order part mainly comprises the S_1_ (or 2D_1_) band (around 2700 cm^−1^) [39]. The G band corresponds to the C–C elongation mode in the aromatic rings, and therefore corresponds to the vibrations of the sp^2^ carbons [40]. 

In contrast, the D bands observed in the first order part have been attributed to in-plane defects and to heteroatoms. The S_1_ band is as an overtone of the D_1_ band, and is related to the stacking order along the c-axis [41]. From the D_1_ and G bands, their full width at half maximum (FWHM) values and intensity ratio (I_D__1_/I_G_) are calculated, which gives information on the extent of structural defects and the graphitisation degree in a series of carbon materials.

#### 2.3.4. Scanning Electron Microscopy

The carbon fibre microstructure was then evaluated by scanning electron microscopy (SEM) (Philips XL30 SFEG, Amsterdam, The Netherlands, equipped with field-enhanced thermionic emission gun operating at 4 kV) for CFs before and after chemical reaction with molten salts. The SEM micrographs of different samples were used to determine the alterations of the carbon fibres relative to the corresponding initial materials. Measurements of the mean fibre diameter (assuming circularity) were carried out for at least 10 fibres at five different locations in selected CFs, leading to a standard error of less than 10%, and thereby ensuring representative average values.

#### 2.3.5. Textural Properties of CFs

The textural properties of the materials and their modifications were measured by gas adsorption analysis. All the samples were first degassed under vacuum for at least 48 h at 110 °C. Next, a manometric method was carried out by injecting a purified gas into a cell of known volume, already containing a known amount of adsorbent material. The nanoporous structure of the pristine and chemically-reacted CFs were derived from nitrogen (N_2_) and carbon dioxide (CO_2_) adsorption at −196 °C and 0 °C, respectively, performed with automatic adsorption analysers ASAP 2020 and ASAP 2420 (Micromeritics, Norcross, GA, USA), respectively.

Using Microactive^®^ and SAIEUS^®^ software (version 3.0, Norcross, GA, USA), the following parameters were calculated by application of the 2D non-local density functional theory for the heterogeneous surface of pore walls (2D Non-Localized Density Functional Theory (NLDFT)-HS) to N_2_ and CO_2_ isotherms: specific surface area, S_NLDFT_ (m^2^ g^−1^); total pore volume, V_tot, 2D-NLDFT HS_ (cm^3^ g^−1^); micropore volume, V_μ, 2D NLDFT-HS_ (cm^3^ g^−1^); and mesopore volume, V_meso_ (cm^3^ g^−1^) = V_tot, 2DNLDFT-HS_ − V_μ, 2D NLDFT-HS_.

The 2D NLDFT-HS model, rather than the traditional BET model, was used here because it improves the modelling by taking simultaneously into account the adsorption results of nitrogen (N_2_) and carbon dioxide (CO_2_), and considering carbon pore walls with heterogeneous surfaces. Additionally, this modern method combines all pore widths to give a more accurate pore size distribution, thus better representing carbonaceous materials with a poorly developed porous structure and a low surface area [42].

## 3. Results and Discussion

### 3.1. CFs Elemental Composition

In addition to differences in manufacturing processes and precursors [31], CFs also differ in terms of carbon and heteroelement contents. The performance of the analysis showed that the elemental composition of commercial CFs is highly repeatable, with maximal standard deviations of 0.45 wt % for C and O, or 0.01 wt % for the other heteroatoms. As no sulphur was found, this element was omitted from the discussion of the results, given in Table 2 in units of wt %. As expected, C was by far the most abundant element in as-received commercial materials, with minimum values of 76 wt % and 95 wt % for ex-Rayon CFs and ex-PAN CFs, respectively. The carbon amount was further increased when the CFs derived from a given precursor were manufactured at a higher temperature. Oxygen was the second most abundant element, with a maximum value of 18.4 wt % for the ex-Rayon CFs and a value as low as 0.6 wt % for the ex-PAN CFs. A meaningful reason for the presence of O and N is that the regenerated cellulose (Rayon) fibrils are rich in O, and that N can also be originally present as a production impurity, whereas PAN contains large numbers of C≡N functionalities [43,44,45]. Compared to the C content, the amounts of H were found to be low and variable from one material to another, but decreasing again with the increase in the final heat treatment temperature. In general, pyrolysis in inert atmosphere controls the increase of the carbon content through several complex elimination processes of CH_4_, H_2_, N_2_, HCN, H_2_O, CO, CO_2_, NH_3_, tars and other volatiles [46,47,48].

A complementary elemental analysis allowed comparing as-received CFs with de-sized CFs. In the case of all ex-PAN CFs, no significant changes were observed in terms of elemental composition. However, in some cases of carbonised, ex-Rayon CFs (CR1 and CR2), weight losses (averaged on a dry basis) of 7% and 8%, respectively, were observed after the de-sizing treatment. This could produce a slight decrease of O content and an increase of C content. Another exception is the graphitised, ex-Rayon GR1, for which an increase in C content and a decrease in O content were seen, without clear net weight loss. These observations of changes in the elemental composition and weight losses evidence the sizing [49] or any other post-production modification of some CFs, and confirm the need to proceed to an additional de-sizing step before performing all characterisation analyses and chemical reactivity tests.

**Figure 2:**
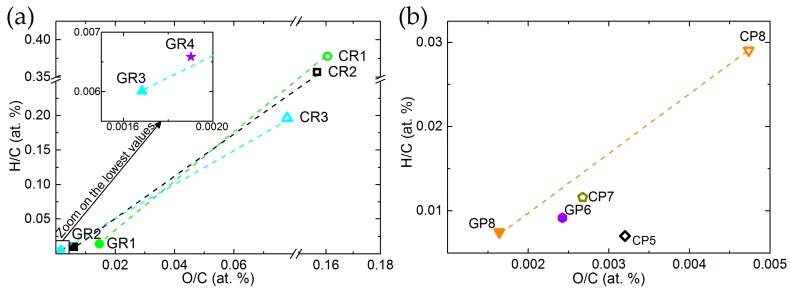
Van Krevelen diagrams of: (**a**) ex-Rayon and (**b**) ex-polyacrylonitrile (PAN) CFs. The dashed lines connect CFs before (empty symbols) and after (full symbols) graphitisation of the CFs.

Van Krevelen diagrams of ex-Rayon and ex-PAN CFs confirm that both O/C and H/C ratios decrease, and that the degree of carbonisation increases with the final treatment temperature (see Figure 2a,b, respectively). In general, most ex-PAN CFs present lower O/C and H/C ratios than ex-Rayon CFs, due to the different elemental composition of the precursors. It can be noted that the y-axis is used to represent the H/C ratios, whereas the x-axis describes the variation of the O/C ratio, with significant differences in scales. This indicates that the elimination of O is favoured, but that a higher number of H atoms is still present in the final structure of the materials. This is clearly visible for the graphitised CFs. Thus, the thermal annealing of CFs leads to a sharp drop of ratios for most of the corresponding materials (such as CP8 → GP8; CR1 → GR1; CR2 → GR2; and CR3 → GR3), also suggesting the improvement of the structural and chemical stability of the carbon by decreasing the heteroatom content. Remarkably, it is evident that different precursors or fabrication processes produce CFs having different elemental compositions, while all were produced in an inert atmosphere, and with similar final heat-treatment temperatures.

### 3.2. CFs Nanostructure

#### 3.2.1. XRD Analysis

**Figure 3:**
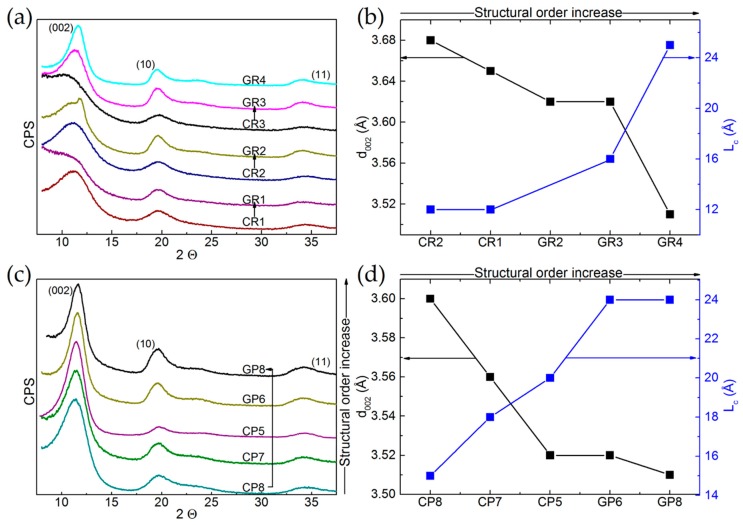
X-ray diffraction (XRD) patterns of: (**a**) ex-Rayon CFs, and (**c**) ex-PAN CFs, presented as a function of the gradual increase in their structural order. The arrows connect corresponding CFs, and the patterns are shifted with respect to each other by a constant number of counts per second (CPS, arbitrary units). Corresponding results of interlayer spacing (d_002_) and nanocrystallite thickness (L_c_), plotted against increasing structural order for: (**b**) ex-Rayon CFs, and (**d**) ex-PAN CFs.

The XRD patterns of the 12 commercial materials are given in Figure 3a,c for ex-Rayon CFs and ex-PAN CFs, respectively, and these are presented as a function of the gradual increase in their structural order. Figure 3b for ex-Rayon and Figure 3d for ex-PAN CFs relate the characteristics of the (002) reflection: position, width and intensity, to the extents of interlayer spacing (d_002_) and of graphitic layers’ stacking (L_c_) using Equations (1) and (2), respectively.

The (002) reflections of the ex-Rayon, carbonised CFs appear very broad and not completely defined, especially for the materials CR1, CR2 and CR3, because of the large number of highly disordered carbon regions. Their low structural order results from semi-crystalline conformations of the precursor, which have been found to be connected with disordered transition regions containing heteroatoms, thus limiting rearrangement during pyrolysis processes [50,51]. As for the corresponding GR1, GR2 and GR3, an absence or a partial improvement of the structural order was observed after the graphitisation step (above 2000 °C). The higher heat-treatment temperature clearly defined these materials as non-graphitising (GR1) or partially-graphitising (GR2, GR3). The splitting of the (002) reflection suggests a structural heterogeneity of the partially-graphitising material (clearly seen for GR2), due to the presence of graphitic nanocrystallites of different sizes and different interlayer spacing [52]. The graphitised GF4 gave narrower reflections slightly shifted to a higher Bragg angle. The performance of pyrolysis at low heating rates and the use of impregnants or organosilicon compounds (stabilisers) [53,54] can explain the structural improvements with the highest evaluated structural order. In general, the XRD patterns of the graphitised, ex-Rayon CFs also suggest the absence of a hot stretching step at the late carbonisation stage in the manufacturing process of the carbon fibre [53], crucial for the development or improvement of the carbon structural order. The diffraction patterns obtained from ex-PAN CFs prepared at temperatures below 1200 °C exhibited a better ordered carbon structure than that of carbonised, ex-Rayon CFs. Graphitised, ex-PAN CFs are materials with improved turbostratic structure, resulting in sharper and narrower (002) reflections [45] compared to carbonised CFs. However, no tri-periodic stacking formation of graphitic structure is observed in ex-PAN or ex-Rayon CFs, according to the low definition or the absence of (hkl) reflections. Moreover, the discussion of the structural properties of CFs is omitted with respect to two-dimensional XRD reflections (hk0) because of their poor appearance and the possibility of important estimation errors [55,56]. 

The samples having a very poor structural arrangement produce broad (002) diffraction bands of asymmetrical appearance (CR3, GR1 and, to a certain extent, GR2), the deconvolution of which did not result in accurate fits. When they could be clearly deconvoluted, the (002) reflections were used to calculate the values of structural parameters d_002_ and L_c_. The values of d_002_ and L_c_ decrease and increase, respectively, for materials having a higher structural organisation and alignments of graphite nanocrystallite layers. In general, d_002_ values are observed in the range from 3.5 to 3.68 Å for ex-Rayon CFs, and from 3.5 to 3.6 Å for ex-PAN CFs, i.e., these are much higher than those of graphite, 3.35 Å [57]. Therefore, the low structural order and the absence of tri-periodic graphitic stacking have been confirmed for these fibrous carbons, assumed to be of general use because they are produced without advanced methods of structural improvement.

**Figure 4:**
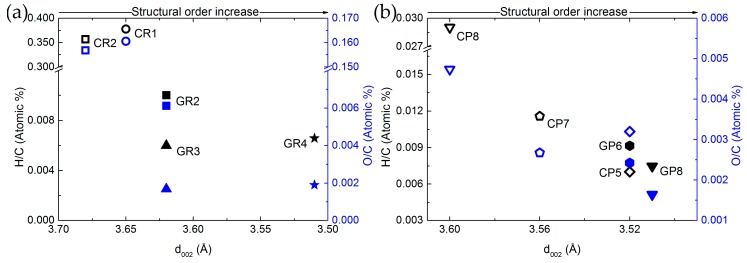
Elemental composition as a function of interlayer spacing (d_002_) of: (**a**) ex-PAN CFs, and (**b**) ex-Rayon CFs. Empty symbols represent carbonised CFs and full symbols represent graphitised CFs. The difference in colour corresponds to O/C (blue) and H/C (black) atomic ratios.

Comparing elemental compositions and structural properties was then performed to obtain additional cross-property relationships. For this purpose, Figure 4 shows the O/C and H/C atomic ratios as a function of the interlayer spacing (d_002_) for ex-Rayon and ex-PAN CFs.

There is a reasonable correlation between the decrease of H and O contents with respect to that of C, and the increase of structural order. In general, d_002_ decreases when reducing the amount of heteroatoms in the carbon lattice, which allows additional arrangement within the graphitic layers. These observations confirm that the structural information derived from the (002) reflection also reflects the degree of carbon purity in the CFs, indicated previously for other carbons [58,59]. The ex-Rayon CFs have slightly scattered results, but still follow the same trend as ex-PAN CFs. The corresponding pairs of CFs from the two precursors, i.e., CR2 vs. GR2 and CP8 vs. GR8, show a very good correlation between the elemental composition and the properties of the graphitic nanocrystallites. Moreover, for both ex-PAN CFs and ex-Rayon CFs, the impact of the structural properties of the precursor and the preheating treatment steps is clearly observed for carbonised CP5 and for graphitised GR2 and GR3, respectively. Although the carbonised CP5 sample shows comparable or higher carbon purity and structural properties than many graphitised CFs, the latter samples have a high purity, but only a partially or completely non-graphitic structure. As a result, a moderate structural improvement is often observed for biosourced carbon fibres, even after having been subjected to high-temperature treatments [52,53,60].

#### 3.2.2. Raman Spectroscopy Analysis

**Figure 5:**
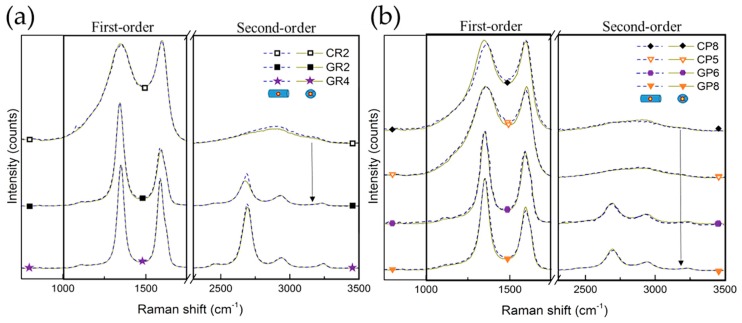
Raman spectra of: (**a**) ex-Rayon CFs and (**b**) ex-PAN CFs, where the arrows connect the corresponding CFs. The dashed or solid lines represent spectra of outer fibre surface or cross-section of carbon fibres, respectively. For easier viewing, an identical intensity shift has been applied.

After carrying out the overall structural XRD analysis, Raman spectroscopy was performed for local structural studies of selected CFs. Random zones on different carbon fibres were examined from the outer surface or cross-sections of the fibres (presented in Figure S1). Figure 5 shows the Raman spectra of the studied CFs, shifted with respect to each other for easier visualisation of the similarities or differences. Moreover, the Raman spectra obtained from the same CFs at the cross-section (dashed lines) and the outer fibre surface (solid lines) are completely superimposed, suggesting structural homogeneity. A first-order part is seen (in the range from 1000 to 1800 cm^−1^) presenting an intense and broad D band (around 1350 cm^−1^) and a slightly narrower G band (around 1590 cm^−1^). A second-order part (in the range from 2200 to 3400 cm^−1^) is also observed, with various degrees of structuration, depending on the investigated CFs. Therefore, clear differences in spectra are observed when comparing the corresponding CFs, with the only difference being in the final manufacturing treatment temperature, as seen for CR2 and GR2 and for CP8 and GP8 (linked by arrows).

From the deconvolution of the Raman spectra (see Figures S2 and S3), the features of carbonised CFs are considered very typical of highly disordered carbon fibres [60,61,62,63]. Fabricated with additional heat treatment, the graphitised CFs have narrower first-order bands, with a difference in band intensity and a much deeper valley between D_1_ and G bands. 

The bands D_1_, D_3_ and D_4_ correspond to in-plane defects, out-of-plane defects and impurities in the sp^2^ graphitic network, respectively [40], whereas the G band solely represents the stretching vibration in the aromatic layers of graphite-like carbon [59]. The constant presence of the G band in the examined CFs explains the clear existence of sp^2^-hybridised carbon, and the intensity and width of this band are both influenced by the three dimensional (3D) evolution of the graphitic nanocrystallites. It is also found that the D_2_ band, observed only for graphitised CFs, is related to surface graphene sheet vibrations, and can therefore be used as a possible indicator of the surface-to-volume ratio of crystallites in nanocrystalline, graphitic carbons [39]. Changes in these bands result from the improvement of the structural order of the materials, accompanied by the decrease of D_4_ and D_3_ and the appearance of the D_2_ band. The presence of D_1_ in all CFs samples indicates sp^3^-hybridised carbon atoms, as such or more probably related to the presence of oxygen, hydrogen and/or nitrogen in the CFs, in good correlation with the results of elemental analysis. In addition to the structural disorder, the D_1_ band also comes from the presence of small graphitic nanocrystallites with a low number of graphitic layers, confirmed by the results of XRD.

**Figure 6:**
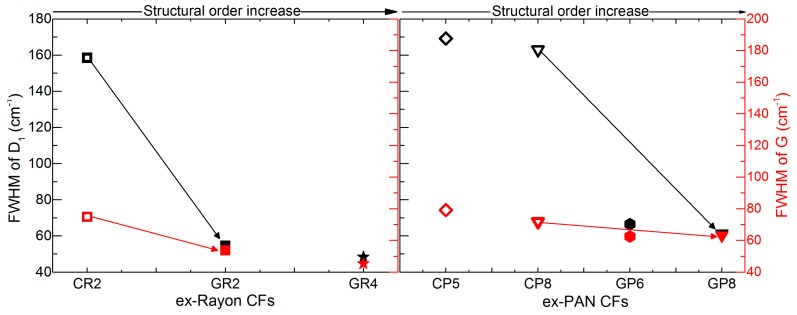
Full width at half maximum (FWHM) of deconvoluted D_1_ and G bands of ex-Rayon CFs (left) and ex-PAN (right) CFs, where the arrows connect the corresponding CFs. Empty symbols represent carbonised CFs and full symbols represent graphitised CFs, the colour differences corresponding to D_1_ (black) and G (red).

The same kind of conclusion is obtained from the estimated full width at half maximum (FWHM) values of the deconvoluted D_1_ and G bands, ranging from 169 to 48 cm^−1^ and from 79 to 45 cm^−1^, respectively (see Figure 6). The corresponding pairs of CFs (from the two precursors) show a very good correlation of the FWHM of the D_1_ and G bands with the increase of structural order, comparing CR2 and CP8 with GR2 and GR8, respectively. However, even the lowest FWHM (GR4, at 45 cm^−1^), is much higher than that of highly-oriented graphite, about 15–16 cm^−1^ [40]. Therefore, the analysis indicates a low degree of local graphitic order in the examined CFs, which confirms the validity of the d_002_ and L_c_ values from the XRD analysis.

When the band deconvolution is performed for the second order of the Raman spectra, a clear appearance of the intense S_1_ band (attributed to the first overtone of D_1_ at around 2700 cm^−1^) has been found only for graphitised CFs. The observed intensity increase of the S_1_ band (as in GR4) should correspond to the lateral growth of the graphitic nanocrystallites, and to the improvement of the stacking order of the graphitic layers [41,60]. However, it is known that the S_1_ band splits into two bands when the structure acquires a tri-periodic stacking order of the carbon layers [39,64], which is obviously not the case for any of the studied materials, in agreement with the XRD analysis.

The intensity of the D_1_ band (I_D__1_) is higher than that of the G band (I_G_) for the examined CFs, including the ones (GR2, GR4, GP6 and GP8) manufactured with a higher final heat-treatment temperature, above 2000 °C. For the corresponding CFs pairs (see Figure S4), this I_D__1_/I_G_ ratio increases gradually, which is an additional indication that these CFs are in the carbonisation regime, whereas in the graphitisation regime, one should expect the contrary [38]. Again, it is likely that only lateral graphite nanocrystallite growth occurs with a supposed lack of the tri-periodic stacking of carbon layers.

### 3.3. High-Temperature CFs Chemical Stability in Molten Li-Based Salts

After having presented the results of elemental composition and structural properties, the chemical stability of these commercial CFs is considered below. Many experimental variables, such as precursor, manufacturing technique or temperature, have been observed to influence the structural and textural properties of the CFs, and therefore should affect their chemical stability in molten Li-based salts. Knowledge of the chemical stability of the CFs is needed to identify the best materials to serve as host structures for PCMs for the intended TES application. It should be emphasised that, as discussed in Section 2.2, the chemical reactivity tests were repeated at least twice for each of the many CFs examined, showing a very low standard error of less than 2% on the carbon mass loss (defined as burn-off, B-O (%) = 100 − percent of carbon disappeared by initial weight of CF). Finding a high reproducibility of the reactivity tests makes it possible to compare the CF samples tested in molten LiOH, LiBr and Li_4_(OH)_3_Br, and to correlate the textural and morphological modifications studied.

#### 3.3.1. Chemical Stability of CFs in LiOH with Regard to Their Structural Properties

The chemical stability tests revealed that losses of carbon mass (i.e., B-O) always occurred, and increased with temperature (values indicated in Tables S1 and S2). This is obvious because of the considerable increase in the reaction rate and the existence of surface functional groups, considered as catalysts for the following reaction [35]:

2C + 6LiOH → 2Li + 3H_2_ + 2Li_2_CO_3_ Reaction (1)

**Figure 7:**
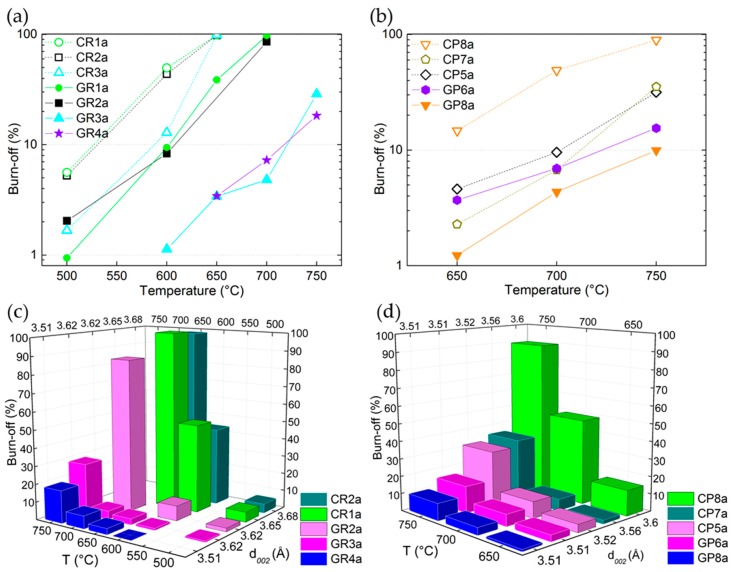
Burn-off (B-O) (%) as a function of reaction temperature for: (**a**) ex-Rayon CFs and (**b**) ex-PAN CFs. Empty and full symbols represent carbonised CFs and graphitised CFs, respectively; the dashed or solid lines are only guides for the eye. Three dimensional (3D) bar representation of changes in burn-off (%) versus test temperature and interlayer spacing (d_002_) for: (**c**) ex-Rayon CFs and (**d**) ex-PAN CFs, tested for chemical reactivity in molten LiOH.

Clearly, for both ex-PAN and ex-Rayon materials, the reactivity of LiOH is reduced as carbon purity increases, and the CFs become very stable even at the highest temperature tested (see Figure 7a,b). This confirms that the rate of reaction (1) and its activation energy can be modified when testing the reaction of LiOH with carbonaceous materials having different graphitisation stages. In general, by comparing only carbonised materials obtained from different precursors, the ex-Rayon CFs show a higher B-O than the ex-PAN CFs, in good agreement with their higher number of heteroatoms. Moreover, the differences in reactivity are clearly observed between the corresponding CFs (CR1a → GR1a, CR2a → GR2a, CR3a → GR3a or CP8a → GP8a). In this context, the presence of O, N and H in the CFs is associated with the existence of surface functional groups (–C–H; –C–OH; –C–O–C; –C=O; C=N; –COOH; etc.), the number of which decreases with subsequent heat treatments at a higher temperature [33,43,45,65,66,67,68,69]. Thus, the B-O results correlate well with the Raman analysis, where the carbonised CFs spectra showed broad and intense D_1_, D_4_ and D_3_ bands, indicating a significant number of heteroatoms and aliphatic side chains.

In addition to the discussed correlation with their elemental composition, the graphitic improvement of the graphitic order of the CFs is also compared to the results of the chemical stability tests. As seen in Figure 7c,d, the values of B-O are generally correlated with the interlayer spacing (d_002_). The plotted results clearly show the downward trend in the CFs’ chemical reactivity in molten LiOH as the structural order increases, but this correlation should be considered with caution. Indeed, some CFs had a similar interlayer spacing (d_002_), such as in GR3a ↔ GR2a and GP8a ↔ GP6a, but with different B-O values. Coming back to Figure 4, a clear indication of this behaviour can be inferred from the different amounts of O and H in the mentioned CFs. Thus, even if a similar structural development is observed, especially for GP8a ↔ GP6a (from both XRD and Raman analyses), the higher number of heteroatoms in the latter clearly distinguished this material as being more reactive with LiOH. A slight difference from the downward trend is also observed for CR2a and CR1a, again in good correlation with the elemental composition. Thus, the current correlations show that LiOH is more reactive (higher B-O at a lower test temperature) in the presence of low-purity CFs. In most cases, this was less dependent on the graphitic order achieved during the last manufacturing process (carbonisation or graphitisation), taking into account CFs from different suppliers. However, the graphitised CFs with improved structural order and higher crystallite size clearly appear as better candidates in the TES application, which should further improve the thermal management properties of the PCM-CF composite materials [70,71].

#### 3.3.2. Chemical Stability of CFs in LiOH with Regard to Their Textural Properties

**Figure 8:**
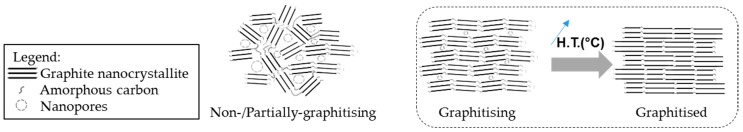
Franklins’ structural models of different carbon nanostructures and the presence of nanopores.

A higher structural order also prevents the development of porosity in carbon fibres, as presented by the Franklin carbon models [72] in Figure 8. The imperfections of the graphitic network are reduced by the development of the graphitic nanocrystallites, thus inhibiting the microporosity.

Therefore, since the chemical stability of CFs has been discussed with respect to their elemental and structural properties, the same correlations have been sought with their nanotexture properties. The textural analysis carried out was based on N_2_ and CO_2_ adsorption isotherms on the initial (de-sized) CFs and those reacted in molten Li-based salts (the detailed results of textural analysis are available in Section S2 of the Supplementary Material). A large variety of nanotextures has indeed been observed for CFs with different structural properties, as shown by the adsorption isotherms of Figures S5–S7. Then, the application of the 2D NLDFT-HS model was chosen as quite advantageous in terms of the improved accuracy of the calculated textural properties. Moreover, the use of an up-to-date calculation model is very important when adsorption equilibrium is difficult to reach during the nanotexture analysis of carbons with heterogeneous and very narrow porosity [73].

**Figure 9:**
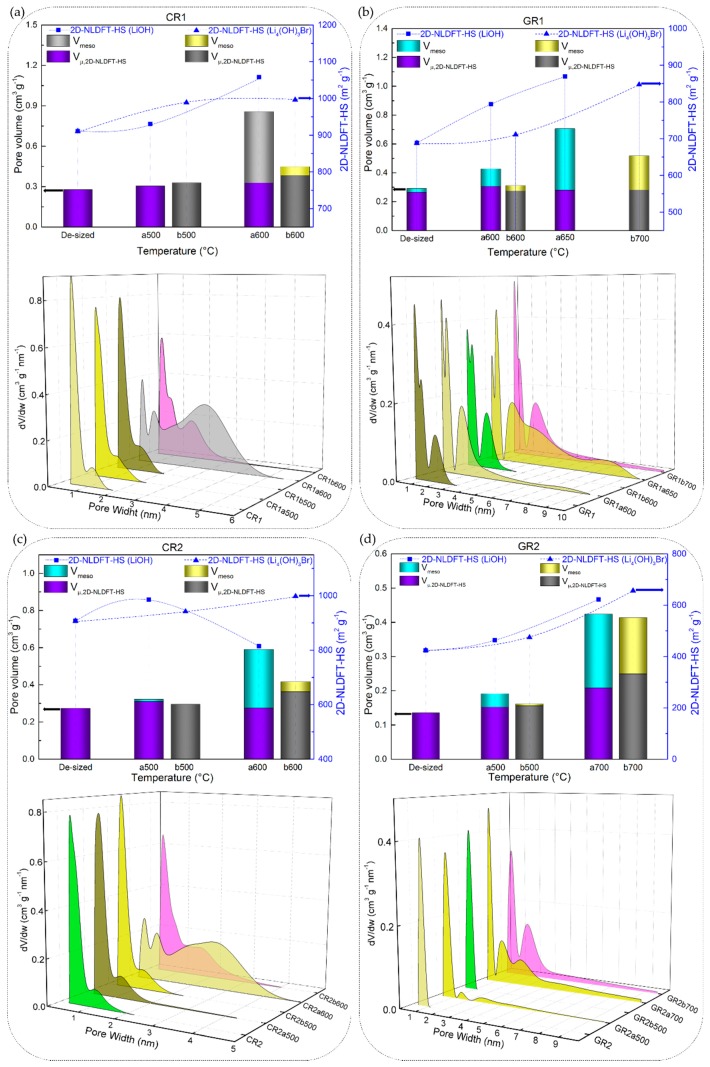
2D Non-Localized Density Functional Theory (NLDFT)-HS textural properties: specific surface area (scatter symbols, where the dotted or dashed lines are only guides for the eye), micro and mesopore volumes (stacked columns), and pore size distributions (3D plots) of corresponding CFs: (**a**) CR1 and (**b**) GR1, (**c**) CR2 and (**d**) GR2, as a function of reaction temperature in molten LiOH or LI_4_(OH)_3_Br.

The 2D NLDFT-HS surface areas obtained from the CFs reacted in molten LiOH were found to vary between 9.6 and 1034 m^2^ g^−1^. Although LiOH showed greater reactivity (given the high B-O values) with carbonised CFs with respect to graphitised ones, the same upward trend was not observed from the results of the 2D NLDFT-HS surface area. As seen in Figure 9a,b,d, CR1a, GR1a and GR2a show a monotonous increase of surface area, at a test temperature of 500 or 600 °C. In addition, CR2a (Figure 9c) is an exception to the monotonic trend with an initial increase (from 907 to 986 m^2^ g^−1^ at 500 °C) followed by a decrease in surface area at 600 °C (814 m^2^ g^−1^). By further increasing the test temperature, the carbonised CR1a and CR2a were completely consumed, and textural analysis was not possible. Whereas, their corresponding graphitised GR1a and GR2a again showed a slight increase of surface area when tested at higher temperatures, 650 and 700 °C, respectively.

**Figure 10:**
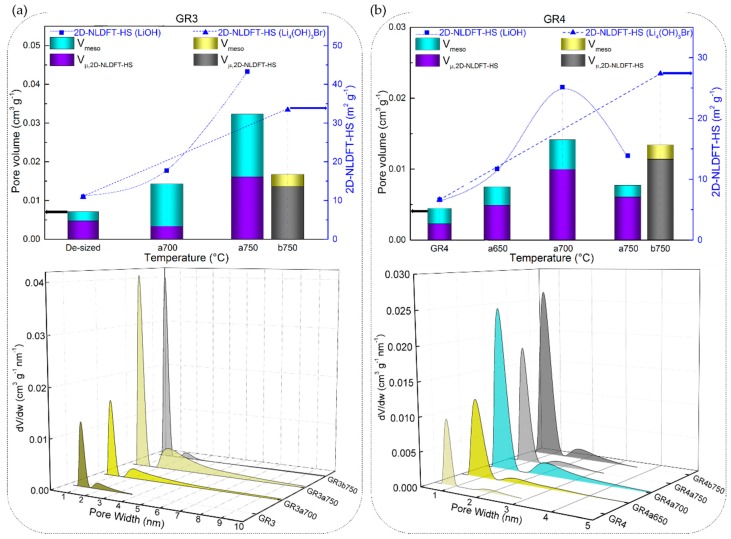
2D NLDFT-HS textural properties: specific surface area (scatter symbols, where the dotted or dashed lines are only guides for the eye), micro- and mesopore volumes (stacked columns), and pore size distributions (3D plots) of graphitised CFs: (**a**) GR3 and (**b**) GR4.

These results of the low development of porosity, or even loss of porosity, together with the high values of B-O, are observed due to the high LiOH/CF molar ratio of 20/1, leading to an intense chemical reaction with the carbon, whose reaction rate increases with temperature. Moreover, a high chemical reactivity in molten LiOH has been reported elsewhere for different carbon materials, even when using a much lower LiOH/C molar ratio of 1.5/1 [74]. Another important feature is the presence and role of amorphous carbon that disorderly crosslinks graphitic nanocrystallites in the carbon fibre structure, leading to the presence of accessible nanopores and heteroatoms. When comparing the carbonised CFs to the graphitised CFs, the fraction of disordered phase is larger, and the very accessible atoms are more likely to perform chemical reactions than the carbon atoms of the graphitic nanocrystallites. The opposite was evident when analysing GR3a and GR4a CFs, as these materials have a significantly higher structural organisation and carbon purity. Even at higher temperatures, 700 and 750 °C, LiOH reacts to a much lesser extent with these CFs, resulting in negligibly low surface areas (see Figure 10a,b).

These results confirm the information of high structural order, deduced from the Raman spectroscopy (as for GR4). In addition, the results are in good correlation with the lower oxygen content, whose presence has been shown to facilitate the fixation of alkaline ions, thus catalysing carbon gasification reactions [75].

The same trends of surface area were observed when analysing the textural properties of the ex-PAN CFs. Likewise, the carbonised CFs (represented by CP8a) showed a higher surface area than the corresponding graphitised GP8a, after being heat-treated in molten LiOH at an identical test temperature. However, textural analysis was performed on only a few reacted ex-PAN CFs, due to the poorly developed porosity and high experimental error risk. The obtained results are presented in Table S2.

In addition, the total pore volume and pore size distributions (PSDs) obtained with the 2D NLDFT-HS model further confirm the observed trends for the specific surface areas. Figure 9 and Figure 10 also show that very different porosities are obtained, in terms of micro-/mesoporous volume, depending on the initial CFs properties and their reactivity in molten LiOH at the temperatures tested. In general, for all CFs examined, the increase in micropore volume occurred only by widening the initial micropores, but also because of the appearance of medium-sized micropores (0.7–2.0 nm) and mesopores, in good correlation with the PSDs. At a lower temperature of 500 °C, the PSDs were slightly modified by widening the medium-sized micropores of the carbonised CFs, due to the slow chemical reaction (see the PSDs in Figure 9a,c). As for the corresponding graphitised CFs (Figure 9b,d), the initial PSD was obviously different due to structural differences, leading to narrower pores. Thus, GR1 had both ultramicropores (< 0.7 nm) and medium-sized micropores, the latter extending their range to mesopores, whereas GR2 was purely microporous, in good agreement with the structural information. When testing the latter two CFs at 500 °C, it was observed that the chemical reaction in LiOH resulted in a widening of the medium-sized micropores in GR1a500, whereas medium-sized micropores and mesopores formed in GR2a500. In both CFs, this clearly influenced the increase in mesopore volume (from 9.0% to 20.5% and from 0.1% to 20.4%, respectively). Increasing the test temperatures led to an increase in the total pore volume in most of the CFs studied. For poorly ordered carbonised CFs (CR1 and CR2), this was mainly due to the significant opening of the microporosity initially observed and the increase in mesoporosity (e.g. from 0.30 to 0.86 cm^3^ g^−1^ for CR1a600, and from 0.23 to 0.59 cm^3^ g^−1^ for CR2a600 at 600 °C). The PSDs of the aforementioned CFs had merged regions of micro- and mesopores, which even caused a clear decrease in the specific surface area of CR2a600. With regard to the corresponding graphitised CFs (GR1 and GR2, respectively) of improved structural order, only the medium-sized microporosity was involved in the development of a higher micro-/mesopore volume without clear evolution of the initial ultramicropores (see Figure 9b,d). Finally, the examined textural results of GR3 and GR4 with higher carbon purity and improved structural order confirm the important effect of the initial properties of the materials on the significant reduction of their reactivity in molten LiOH (see Figure 10a,b).

It is difficult to make an in-depth comparison between the results presented here and those obtained for different carbon materials, because of the different variables used, such as carbon precursor and experimental conditions. In general, the reviewed bibliography is consistent with our results on the chemical reactivity of CFs in molten LiOH and the observed loss of carbon weight, but with poor development of surface area and microporosity [74,76,77,78,79,80,81,82,83]. The main reason is always indicated as being the strong reaction of LiOH under the experimental conditions used and the high ratio LiOH/carbon material. The small size of the alkaline atom should also have promoted high reactivity, allowing easier access to the initial material porosity and further pore widening [84]. This resulted in a higher carbon mass loss in carbonised CFs compared to graphitised CFs. No metallic Li intercalation was observed in any of the CFs examined, as evidenced by XRD and supported by the textural analysis, still showing poor porosity development. This can be explained by the fact that Li cannot be converted to metal vapour (in the temperature range studied, up to 750 °C), then easily diffused and intercalated between the carbon layers [75,84,85,86]. This is in a good agreement with the similar chemical stability of CFs with a different structural order, but with high carbon purity (see Figure 4).

#### 3.3.3. Chemical Stability of CFs in LiOH with Regard to Their Morphology

**Figure 11:**
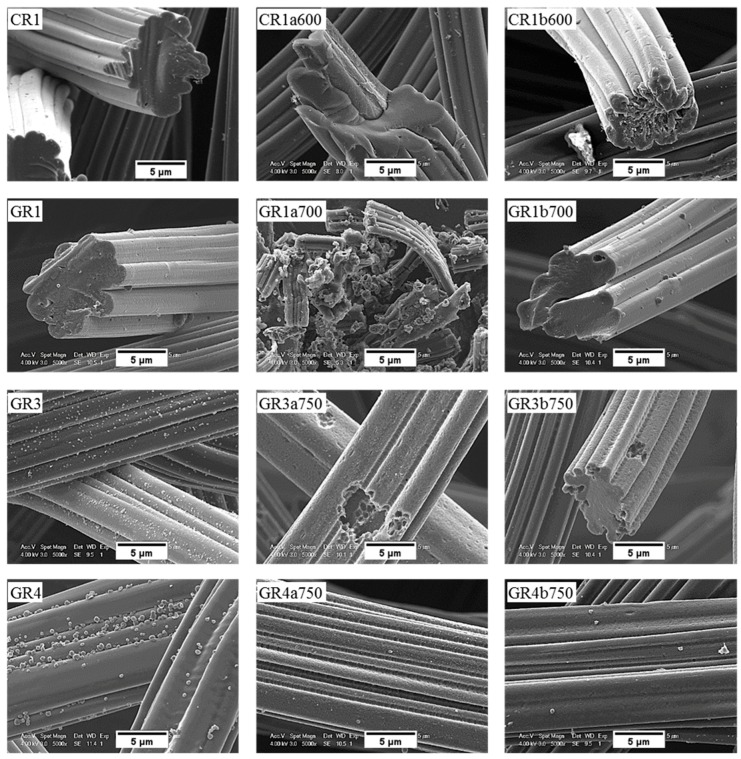
Scanning electron microscopy (SEM) micrograph of de-sized ex-Rayon CFs before (**left**), and after reaction with either LiOH (**middle**) or Li_4_(OH)_3_Br (**right**) at the highest temperature tested.

**Figure 12:**
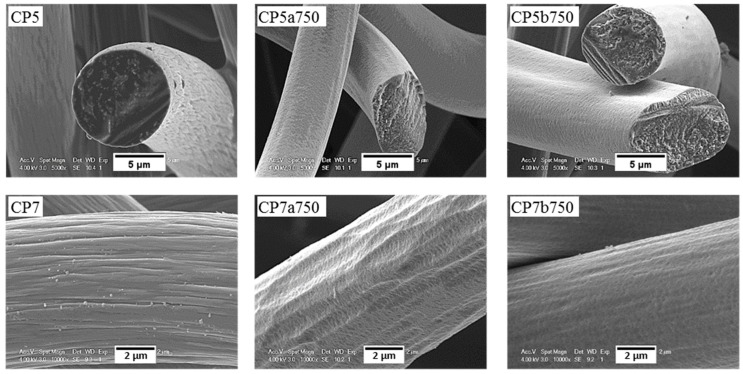
Same as Figure 11, but for ex-PAN CFs.

SEM micrographs of the carbon fibre morphology of de-sized materials before and after reaction in molten LiOH are shown in Figure 11 and Figure 12 for ex-Rayon CFs and ex-PAN CFs, respectively. As detailed in a former study from our group [31], the morphology of the outer surface and the cross-section of de-sized carbon fibres prior to reaction varies with the precursor and the manufacturing technology, since these commercial materials come from different suppliers. 

Moreover, the only observations common to these carbon fibres are grooves and striations parallel to the axis of ex-Rayon fibres and of ex-PAN fibres, respectively, because of the manufacturing process of the fibres’ precursor. The microscopic impurities visible on the surface of most carbon fibres are probably by-products of de-sizing. Ex-Rayon CFs had pores between the constituent fibrils (as seen for GR1), clearly observed from the inhomogeneous cross-section of the carbon fibres. Whereas, ex-PAN CFs showed an obvious absence of these pores both on the outer surface of the fibres or in the cross-section. Therefore, further investigations were conducted to determine how the carbon fibre structure was modified during surface contact with LiOH, either uniformly or in a defined pattern, due to the various characteristics of the different CFs.

Most carbon fibres constituting the CFs retain their original global structure after the reactivity tests. However, some of them have been destroyed due to a severe chemical reaction with LiOH (as GR1a700), in good agreement with the results of B-O at high temperatures. From carbonised ex-Rayon CFs, we observed that the prolonged chemical reaction with LiOH deepened the initial grooves and created new cracks, clearly triggering the longitudinal division of the carbon fibres (as shown by the carbonised CR1a600). 

The same is not clearly observed in the other carbonised CR2a600 and CR3a600 when they reacted at the same temperature, but the destruction of the fibres occurred after the increase in the test temperature (CR3a650) (see Figure S8). This confirms the use of different materials and methods of manufacturing ex-Rayon carbon fibres and their influence on the structure of the final materials, even with similar final heat-treatment temperatures. The influence of the materials’ manufacturing history was also seen when comparing the graphitised ex-Rayon CFs. The sample GR1a700 was almost completely oxidised in the reaction with LiOH, and only indeterminate particles were observed as residues. On the contrary, in the case of samples GR3a750 and GR4a750, even after activation at the highest temperature tested, only pores appear on the initial surface, caused by the etching of disordered carbon zones. Moreover, the core of these latter samples remains unchanged in the reacted carbon fibres, again confirming their higher purity and structural order. Thus, the larger pits observed in GR3a750 relative to GR4a750 can be attributed to the higher fraction of disordered domains, originating from heterogeneities in cellulosic fibres [48]. In addition, the chemical stability of GR1, GR3 and GR4 CFs in LiOH must also be the result of their manufacturing differences from non-, partially- or well-stabilised Rayon fibres, respectively [53]. Therefore, SEM microscopy data confirm structural properties’ results, and ex-Rayon carbon fibres with less disordered domains should always be preferred when high chemical stability of CFs in molten LiOH is required.

For ex-PAN CFs, no swelling or longitudinal peeling of carbon fibres was observed, as was the case for some ex-Rayon CFs or for stabilised PAN fibres chemically activated with different alkali hydroxides at much lower weight ratios and reaction times [87]. Here, only surface etching and smoothening were evidenced, which clearly indicates that LiOH alters the fibre morphology differently due to differences in chemical reactivity. As presented elsewhere [49,56], it was also observed that subsequent carbonisation and graphitisation treatments had an influence on the structural improvement of ex-PAN carbon fibres, which controlled morphological changes during air oxidation and chemical activation in KOH or NaOH. Here, a clear effect of the chemical reactivity in molten LiOH was only observed at higher temperature, above 650 °C. Thus, the studied carbonised ex-PAN CFs (CP5a750 and CP7a750) experienced surface smoothening and a shrinkage of the carbon fibre diameter of 2 µm on average (see Figure 12). On the other hand, the outer surface of the graphitised ex-PAN carbon fibres showed modified grooves and striations, elongated parallel to the fibre axis direction, following that observed in the initial materials (see Figure S9). Again, an overall decrease in fibre diameter of 0.5 µm was observed, but much lower compared to carbonised CFs (by comparing the corresponding CP8a650 and GP8a650). The additional increase of test temperature widened the submicron surface pits, and a larger decrease in fibre diameter was observed due to the increased chemical reaction kinetics (as for GP8a750 and GP6a750).

The chemical stability of the investigated CFs, regardless of their originating precursor (ex-Rayon or ex-PAN), is directly related to the stability and persistence of their graphitic structure. Again, from the SEM analysis, the chemical stability of the CFs is identified when comparing materials of similar crystallites size (L_c_) and interlayer spacing (d_002_), but also similar carbon purity. Both GR4a750 and GF8a750 show only slight surface changes after the chemical reaction in molten LiOH, without damaging the overall structure of the constituent carbon fibres.

#### 3.3.4. Chemical Stability of CFs in LiBr or Li_4_(OH)_3_Br Compared to that in LiOH

**Figure 13:**
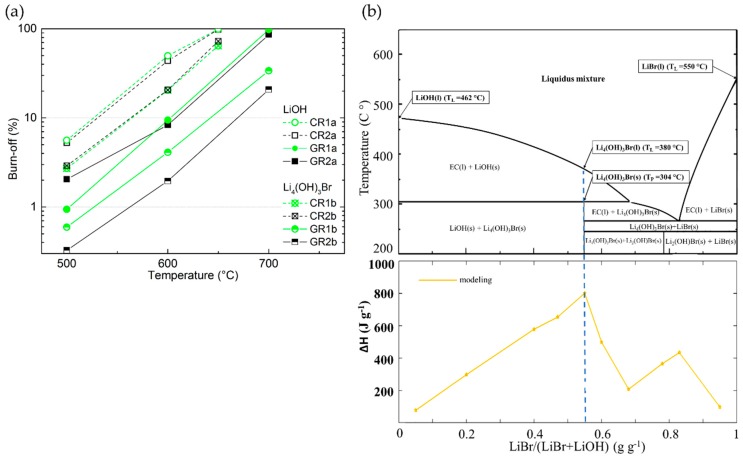
(**a**) Burn-off (%) as a function of reaction temperature of ex-Rayon CFs; empty and full symbols represent CFs reacted in LiOH, and crossed or half-full symbols present CFs reacted in Li_4_(OH)_3_Br; the dashed or solid lines are only guides for the eye. (**b**) Upper figure: Theoretical phase diagram of a LiOH/LiBr binary system, with the corresponding peritectic transition (T_P_) and liquidus temperatures (T_L_); Bottom figure: Gravimetric energy density dependence of the LiBr/(LiBr + LiOH) ratio as seen from the calculated (yellow) line (after [30]).

The study of the chemical stability of CFs in molten LiBr or Li_4_(OH)_3_Br was then carried out in the same way as for LiOH, and compared to it. The results of this section reveal that the nature of the inorganic lithium salts plays a major role in the chemical reactivity of CFs. Thus, in all examined samples (Table S1), a lower chemical reactivity was observed in molten Li_4_(OH)_3_Br with respect to LiOH (visible graphically for the corresponding carbonised and graphitised CFs in Figure 13a with values of B-O 50% lower, or even less. As previously observed for LiOH, it was again found that the chemical reactivity in molten Li_4_(OH)_3_Br is higher for ex-Rayon CFs than for ex-PAN CFs with a similar or lower heat-treatment temperature. Indeed, the main properties that regulate the chemical stability of CFs in molten Li_4_(OH)_3_Br are found to be the purity of carbon and the structural order, in the same way as for LiOH.

In addition, to study the role of the second component of the peritectic compound on the chemical reactivity of CFs, tests were carried out in molten LiBr only for the two CFs (carbonised CR1 and CR2) which proved to be the most reactive with LiOH. The chemical reactivity tests were performed at the highest temperature experienced (750 °C), all other conditions remaining unchanged. 

By repeating the same experiment more than twice, no significant difference in weight before and after the performed treatment was observed. Thus, LiBr alone does not affect the carbon structure of the materials, and its chemical reactivity with the CFs is considered negligible. The chemical inertness of CFs in molten LiBr can be explained by the very high melting temperature of LiBr (about 550 °C) [34]. Moreover, the low reactivity of LiBr–KBr eutectic salts with carbons (or their initial precursors) was previously used to produce 3D carbon structures from biosourced materials under similar heat treatment conditions to those described herein [88]. Therefore, the obtained results indicate that the undesirable chemical reactivity of CFs with Li_4_(OH)_3_Br could only occur because of reactions between the carbon fibres and the hydroxide. The chemical reaction should start even at lower temperature, compared to LiOH alone, since the melting of Li_4_(OH)_3_Br (PC) begins with the formation of the pre-peritectic phase (EC, around 304 °C, see Figure 13b) [30]. The reaction should be further facilitated when the residual solid LiOH is melted at a higher temperature (about 380 °C) in stoichiometric proportion with LiBr, as shown in the theoretical phase diagram of Figure 13b.

The textural properties of the CFs also show less structural changes when submitted to molten Li_4_(OH)_3_Br, compared to molten LiOH (see Figure 9 and Figure 10). Hence, as with the LiOH experiments, the surface area of CFs tested in molten Li_4_(OH)_3_Br tends to increase with the final test temperature, and is again highly dependent on the CFs structural properties. Although the surface area of the CFs has increased as was usually observed with LiOH, it generally comes from different micro/mesopore volume distributions. Therefore, when the CFs are tested in molten Li_4_(OH)_3_Br, the textures remain essentially microporous, whereas those of materials reacted with LiOH are much more mesoporous. This is clearly visible in Figure 9a,c for samples CR1 and CR2 tested at 600 °C. Thus, the strong reaction in molten LiOH destroys most of the micropores, merging them with the developed high mesoporosity, which is observed in molten Li_4_(OH)_3_Br to a much lesser extent. When tests are carried out on CFs with higher carbon purity and structural order (GR3 and GR4), the porosity is less modified in Li_4_(OH)_3_Br with respect to LiOH (see Figure 10). In agreement, the PSDs are more modified for all the materials tested in molten LiOH compared to those in Li_4_(OH)_3_Br (see also Figure 9 and Figure 10). The results with Li_4_(OH)_3_Br also suggest that only initial micropore widening seems to occur at lower test temperatures, but the development of larger mesopores is moderate, and observed only at higher test temperatures (at 600 °C, for carbonised CFs, and above for graphitised CFs).

The SEM micrographs of the tested CFs confirm that the modifications of the carbon fibres depend largely upon the presence and nature of the inorganic salt. Thus, all ex-Rayon CFs tested in molten Li_4_(OH)_3_Br retain the initial fibre structure, and only surface etching and pitting are observed, compared with the severe destruction of the fibres during the reaction with LiOH (see Figure 11). Uniform, smooth and flat surface carbon fibres without the initial surface striations, were observed after reaction of the carbonised ex-PAN CFs in molten Li_4_(OH)_3_Br (see Figure 12). Again, the lower B-O values observed suggest a smaller decrease in fibre diameter compared to the decrease seen in LiOH.

In general, the chemical reactions of CFs in the investigated molten binary peritectic compound, Li_4_(OH)_3_Br, should be considered similar to those in LiOH, but the presence of the LiBr phase only inhibits the severity of the redox reactions with the hydroxide. The reaction of the hydroxide with the active carbon sites should be inhibited only because of the presence of LiBr, no chemical reaction being observed between it and the carbon. The present LiBr phase does not completely prevent the reactions, but delays them differently depending on the structural properties of the CFs and the test temperatures. This is evident when one sees that the PSDs of the same CF tested in LiOH at a given temperature on the one hand, and in Li_4_(OH)_3_Br at a temperature of 50 to 100°C higher, are the same (see Figure 9 and Figure 10). In particular, the consumption of LiOH during the reaction with the CFs shifts the molar ratio of lithium salts in the binary system from its stoichiometric composition (pure Li_4_(OH)_3_Br phase, presented by the blue dashed line in Figure 13b) to a higher concentration of LiBr phase. The continuous change in ratio and enrichment in the LiBr phase, as well as the production of Li_2_CO_3_ (from reaction (1)) must introduce multiple variations of the LiOH–LiBr binary system, ultimately leading to changes in the stoichiometry at the nanoscale. 

As suggested by the adsorption analysis, the LiBr phase might therefore be trapped in the microporosity after the complete consumption of the LiOH phase, and the subsequent reactions of the latter would only cause a widening of the mesopores in the materials tested in Li_4_(OH)_3_Br.

Depending on the chemical stability of the CFs in the LiOH phase within Li_4_(OH)_3_Br, the relevant stoichiometric ratio for the latter PC can be modified to a different degree, thus jeopardising the high initial energy density (see Figure 13b). Therefore, choosing the most inert CFs should inhibit the chemical reaction in molten LiOH at the application temperature, thus ensuring one of the major advantages of using unmodified Li_4_(OH)_3_Br in its composition: its high energy-storage potential.

Our study also shows a beneficial reduction in the chemical reactivity of CFs with the LiOH phase in Li_4_(OH)_3_Br, when compared to LiOH alone. Moreover, the reduction in chemical reactivity can lead to an adjustable textural modification of some CFs with a poorly developed graphitic structure (such as CR1, GR1, CR2 and GR2). The use of such reactive phase/inert phase binary systems can play a dual role of pore modification (through carbon activation) and micropore protection (through the presence of the inert phase) resulting in well-developed micro/mesoporous carbon materials. Indeed, the results obtained here show a potential and beneficial use of the binary inorganic salt system for other fields of research and energy/environmental applications, such as the “salt-templated” carbonisation and synthesis of carbons of tuned porosity [88,89,90].

#### 3.3.5. Estimation of CFs Chemical Stability at the TES Application Temperature

The last part of this study was performed to determine the activation energy of the reactions occurring with CFs in molten LiOH. The main idea of this part is to obtain more information about the results of B-O and to use them to estimate the B-O of the materials at the application temperature (between 300 and 400 °C). Therefore, it is expected that the apparent activation energy will change depending on the properties of the CFs and the chemical reactions that occur in the fixed experimental tests. As discussed in Section 3.3.1, the dominant chemical reaction (1) was expected within the limits of the experiments performed. However, it was previously clear that for some carbons of low structural order and low purity, the carbon/metal hydroxide reaction (1) can be followed by consecutive carbon oxidation reactions within the applied experimental limits [77]:

C + Li_2_CO_3_ → Li_2_O + 2CO Reaction (2)

Although Li_2_CO_3_ has a high melting temperature of about 730 °C [91], earlier decomposition can be observed at lower temperatures and even under a constant flow of inert gas, especially in contact with carbons of low graphitisation stage and bearing a high number of surface groups [92]. Therefore, reaction (2) is expected due to the decrease of the activation energy of Li_2_CO_3_ as a product of reaction (1), and its subsequent reaction with unreacted carbon. However, even if it occurs at about 730 °C or less, the rate of reaction (2) is initially low and quite negligible below 800 °C in the presence of a less reactive carbon material containing not more than 10% of foreign elements [93]. In our results, this is seen by the higher B-O and the dramatic change in the textural properties of CFs, with clear destruction of microporosity (as for CR1a600 and CR2a600 seen in Figure 9a,c), both generally seen above 600 °C. However, for all the CFs studied (under fixed test parameters, i.e., by changing only the material), an exponential increase of the B-O is evident by performing the experiment at a higher final temperature. These results clearly show that the kinetics of the chemical reactions that occur depend on the temperature, which makes it possible to use the Arrhenius law to estimate the apparent activation energy. The Arrhenius equation reads as follows:

(3) $$k=Ae^{-E_{a}/RT}$$

where *k* is the reaction rate, *T* is the absolute temperature, *A* is a pre-exponential factor, *E_a_* is the apparent activation energy of the reaction, and *R* is the universal gas constant (8.314 J mol^−1^ K^−1^). As in other studies dealing with carbons oxidation reactions [94], in which the reaction rate constant was simply calculated as the inverse of the test temperature, we implicitly assume that first-order kinetics applies to most of the chemical reactivity process. 

Although the present redox reactions can be expected to occur through an elementary mechanism, a combination of several mechanisms cannot be ruled out from the results obtained. However, previous studies of carbon reactions with oxygen or other catalytic agents [93,95] have shown that the pre-exponential factor and the apparent activation energy change in the same way, even when several reactions occur. This gives the possibility of using the Arrhenius law to obtain a consistent set of comparative values when considering different carbon materials. Therefore, the Arrhenius law is used to calculate the corresponding activation energy, thus determining the B-O of the CFs adjusted to a different temperature, by perfectly applying the linearised form of Equation (3), as follows:

(4) $$\ln(k)=\frac{-E_{a}}{R}\left(\frac{1}{T}\right)+\ln\left(A\right)$$

**Figure 14:**
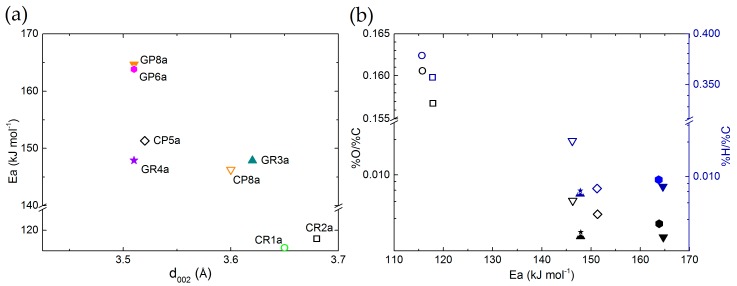
Correlation trends between estimated apparent activation energy and CFs properties, as a function of (**a**) interlayer spacing (d_002_) or (**b**) materials elemental composition. Carbonised or graphitised CFs are represented by empty or solid symbols, respectively.

The application of Equation (3) led determination coefficients (R^2^) higher than 0.919 (see Figure S10). It is found that the apparent activation energy is in the range of 116 to 165 kJ mol^−1^, from carbonised to graphitised CFs, respectively. The carbonised CFs, manufactured at temperatures below 1200 °C, with a highly disordered structure, high porosity (surface area) and active surface (such as surface heteroatoms), were more reactive and more prone to oxidation, even at lower temperatures. For thermodynamic reasons, the existence of an active carbon surface induces a higher number of reactive centres that decrease the activation energy of the reaction [95]. The relationship between all the CFs’ characteristics controlling the chemical reaction justifies the reason for which different slopes were found when fitting the experimental results. Therefore, the apparent activation energy is lower for the carbonised CFs than for the graphitised CFs, with an improved structure and a lower number of surface defects and higher amount of heteroatom content. Thus, it is found that the estimation of activation energy values corresponds to the differences of CFs structure. This is observed when the activation energy values are plotted as a function of the interlayer spacing (d_002_) or the materials’ elemental composition (see Figure 14a,b, respectively). The results follow a consistent order, with a minor scattering of the observed trends due to possible experimental or subsequent calculation deviations.

Furthermore, based on the performed bibliographic research, no activation energy values are reported for similar carbon materials tested under the current test conditions and in molten LiOH. However, the estimated values of apparent activation energy are in good agreement with others for carbon gasification in the presence of Li salts (such as Li_2_CO_3_ and LiOH) under inert atmosphere or in the presence of oxygen, carbon monoxide/dioxide, or in steam [93,96,97,98]. In addition, Cuesta et al. (1993) [95] observed that ex-PAN high-purity carbon fibres react with oxygen at similar apparent activation energy values of between 117 and 134 kJ mol^−1^. 

**Figure 15:**
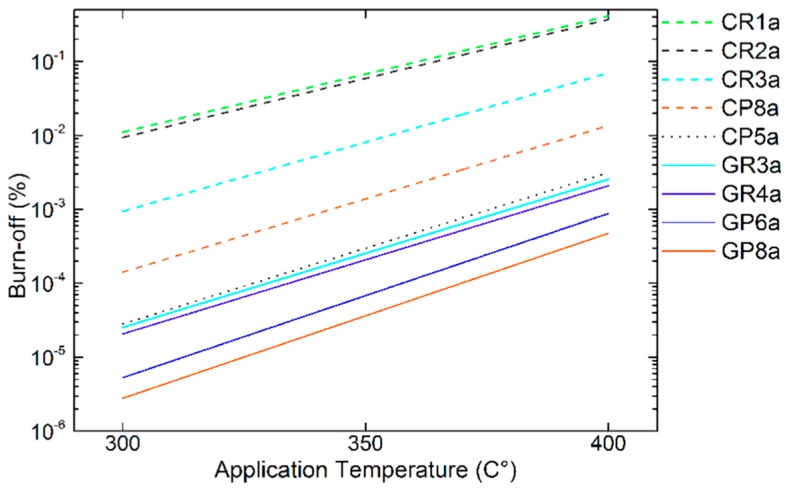
Estimated trends in B-O from the Equation (4), for different CFs, in the application temperatures of interest. Carbonised or graphitised CFs are presented with dashed (ex-Rayon)/dotted (ex-PAN) or solid trend lines, respectively.

Thus, the slightly lower activation energy of high-purity carbon fibres, compared to the present results, was evident because it is known that the reaction of oxygen with carbon is quite exothermic, which decreases the activation energy, unlike CO_2_, steam or other chemical activating agents [99]. Once again, in all the studies reviewed, it was considered that the structural characteristics of the carbons play an important role in the trends in chemical reactivity. Therefore, the calculated parameters related to the chemical reactions that occur in the CFs’ stability tests seem to correlate well with the bibliography. Hence, the subsequent use of the derived experimental results is considered valid for estimating the level of B-O, due to an undesirable chemical reaction between the CF hosts and LiOH at the application temperature (presented in Figure 15).

The set of CFs analysed is extremely diverse, so that materials with different structural properties allow meaningful estimates of reactivity at the application temperature (see Figure 15). The estimated trends of B-O are in agreement with the experimental results, distinguishing the graphitised CFs (i.e., with an improved purity and structure, e.g. ex-Rayon, GR4, and ex-PAN, GP6 and GP8), from the rest. In addition, we must consider that these estimates are based on the results of B-O only with LiOH and that a larger decrease of reactivity is expected with Li_4_(OH)_3_Br, as discussed in Section 3.3.4. Therefore, only those candidates that are chemically most stable and found to be suitable will be considered for the preparation and testing of future CF–PC hybrid materials.

## 4. Conclusions

In this work, we discussed how the structural and textural parameters of commercial fibrous carbons influence their chemical stability in Li- based inorganic salts and promote their use as carbon hosts in TES application. We focused on commercial CFs as mass-produced materials using well-known techniques, considered here as cost-/performance-effective solutions for hosting phase-change materials. The application of different characterisation techniques, including elemental analysis, XRD, Raman spectroscopy, gas adsorption and SEM, allowed us to evaluate clearly the range of properties of the examined CFs, which affected their stability during the chemical reaction tests.

For both ex-PAN and ex-Rayon materials, as carbon purity and structural order increase, the presence of nanopores is reduced, and CFs become chemically stable and fully retain their fibre morphology when in contact with molten LiOH, even at the highest temperature tested. The chemical stability tests confirmed the effect of temperature by increasing the B-O due to the increase in the chemical reaction rate. 

Thus, the main reasons for the observed carbon loss are indicated as being the high LiOH/CF ratio, and the reaction of LiOH with the surface of carbon fibres under the used experimental conditions.

The effect of the salts’ nature was evaluated by performing additional CFs’ chemical stability tests in LiBr or Li_4_(OH)_3_Br. The results show the complete CFs’ inertness in molten LiBr, and increased stability in molten Li_4_(OH)_3_Br, compared to the higher chemical reactivity in molten LiOH alone. In general, the chemical reactions of CFs in Li_4_(OH)_3_Br are considered similar to those in LiOH alone, but the presence of an inert LiBr phase plays a beneficial role in inhibiting the severity of redox reactions with the hydroxide. Finally, it was confirmed that the experimental results of chemical stability tests are in excellent agreement with the values of activation energy estimated by use of the Arrhenius law, distinguishing again graphitised CFs, with improved purity and structure, from the rest.

Finally, the criteria for selecting potential candidates for the TES application based on the peritectic compound Li_4_(OH)_3_Br hosted in fibrous carbon matrices, i.e., CFs with high chemical stability in contact with Li salts, may be obtained from the information presented in this study. In addition, the identification of the critical properties of CFs is a useful guide to further improve materials, but also the use of the CFs and Li_4_(OH)_3_Br in different applications.

## Figures and Tables

**Table 1:** Sorting of the carbon felts (CFs) investigated here into groups, depending on their main characteristics.

Final Heat Treatment	Precursor	Commercial Name	Used Sample Code
Carbonised	Rayon	Carbon (Rayon) felt CeraMaterials	CR1
Graphitised		Graphite (Rayon) felt CeraMaterials	GR1
Carbonised		RSF1 Beijing Great Wall Co.	CR2
Graphitised		RSF2 Beijing Great Wall Co.	GR2
Carbonised		SIGRATHERM^®^ KFA5	CR3
Graphitised		SIGRATHERM^®^ GFA10	GR3
Graphitised		GF2 Schunk	GR4
Carbonised	PAN	PX 35 ZOLTEK^TM^	CP5
Graphitised		GFE-1 CeraMaterials	GP6
Carbonised		BESF Beijing Great Wall Co.	CP7
Carbonised		Carbon (PAN) felt CeraMaterials	CP8
Graphitised		Graphite (PAN) felt CeraMaterials	GP8

**Table 2:** Elemental analysis results (in wt %) of as-received and de-sized CFs.

Sample Code		As-Received CFs				De-Sized CFs			
		C (%)	N (%)	O (%)	H (%)	C (%)	N (%)	O (%)	H (%)
Ex-Rayon CFs	CR1	75.95	3.46	18.37	2.14	77.66	3.18	16.63	2.45
	CR2	77.55	3.17	17.15	2.07	78.45	2.77	16.40	2.33
	CR3	90.94	0.50	7.64	0.92	89.16	0.12	9.27	1.46
	GR1	91.27	0.35	7.46	0.92	97.83	0.13	1.92	0.12
	GR2	98.70	0.43	0.78	0.09	99.01	0.10	0.81	0.08
	GR4	99.53	0.01	0.39	0.07	99.66	0.03	0.25	0.05
	GR3	99.66	0.04	0.24	0.07	99.72	0.01	0.22	0.05
Ex-PAN CFs	CP5	96.82	2.82	0.31	0.05	97.01	2.52	0.41	0.06
	CP8	95.07	4.09	0.65	0.19	95.19	3.91	0.60	0.23
	CP7	97.89	1.59	0.46	0.06	97.85	1.71	0.35	0.09
	GP6	99.46	0.03	0.44	0.06	99.58	0.02	0.32	0.08
	GP8	99.44	0.04	0.46	0.06	99.68	0.04	0.22	0.06

## Supplementary Materials

The following are available online at https://www.mdpi.com/1996-1944/12/24/4232/s1, Section S1: Raman spectroscopy analysis; Figure S1: Raman laser spot position (yellow midpoint), for achieving Raman spectra for both cross-section (left) and surface (right micrographs) of ex-PAN (CP8: top row) and ex-Rayon (GR4: bottom row) carbon fibres; Figure S2: Deconvoluted Raman spectra of ex-Rayon CFs, where the scatter curves indicate the measured intensities and the solid smooth lines represent the fits; Figure S3: Same as Figure S2 but for ex-PAN CFs; Figure S4: Intensity ratio between deconvoluted D_1_ and G bands of corresponding: (**a**) ex-Rayon and (**b**) ex-PAN CFs, with an arrow indicating the increase of structural order. Empty and full symbols stand for carbonised CFs and graphitised CFs, respectively; Section S2: Textural analysis; Figure S5: (**a**,**b**) N_2_ adsorption isotherms at −196 °C, and (**c**,**d**) CO_2_ adsorption isotherms at 0 °C for de-sized CFs before reaction in molten Li-based salts; Figure S6: N_2_ adsorption isotherms of corresponding carbonised and graphitised CFs before and after chemical reaction with LiOH (black symbols) and with Li_4_(OH)_3_Br (green symbols). The arrow is shown for CFs reacted at a same temperature but in different molten salts; Figure S7: Same as Figure S6 but for graphitised ex-Rayon CFs (of highest structural order); Table S1: Textural parameters from both N_2_ and CO_2_ isotherms calculated by 2D NLDFT-HS model of ex-Rayon CFs examined before and after chemical reaction in molten LiOH (normal style) or Li_4_(OH)_3_Br (underlined style). The bold style distinguishes graphitised CFs from carbonised CFs; Table S2: Same as Table S1 but for ex-PAN CFs; Section S3: Scanning electron microscopy; Figure S8: SEM micrographs of de-sized ex-Rayon CFs before (left) and after (middle and right) reaction with LiOH at different temperatures; Figure S9: Same as Figure S8 but for de-sized ex-PAN CFs; Section S4: Estimation of CFs chemical stability at the TES application temperature; Figure S10: Application of Equation (4) to the experimentally derived B-O data of most CFs shown in Figure 7a,b). Carbonised or graphitised CFs are represented by empty or solid symbols and dashed or solid linear fits, respectively.
