# Supplementary material for: Structural Characterisation and Chemical Stability of Commercial Fibrous Carbons in Molten Lithium Salts
Source: Materials (Basel). 2019 Dec 17;12(24):4232. doi: 10.3390/ma12244232 (PMC6947428; doi:10.3390/ma12244232)
Supplement: Supplementary file 1 [file materials-12-04232-s001.pdf]

materials

Article

Structural Characterisation and Chemical Stability of Commercial Fibrous Carbons in Molten Lithium Salts

Blagoj Karakashov [1], Vanessa Fierro [1], Sandrine Mathieu [2], Philippe Gadonneix [1], Ghouti Medjahdi [2] and Alain Celzard [1,*]

[1] Institut Jean Lamour, Université de Lorraine, CNRS, IJL, F-88000 Epinal, France; blagoj.karakashov@univ-lorraine.fr (B.K.); blagoj.karakashov@univ-lorraine.fr (V.F.); philippe.gadonneix@univ-lorraine.fr (P.G.)

[2] Institut Jean Lamour, Université de Lorraine, CNRS, IJL, F-54000 Nancy, France; sandrine.mathieu@univ-lorraine.fr (S.M.); ghouti.medjahdi@univ-lorraine.fr (G.M.)

* Correspondence: alain.celzard@univ-lorraine.fr; Tel.: +33–372–74–96–14.

Supplementary Information:

Section 1. Raman spectroscopy analysis

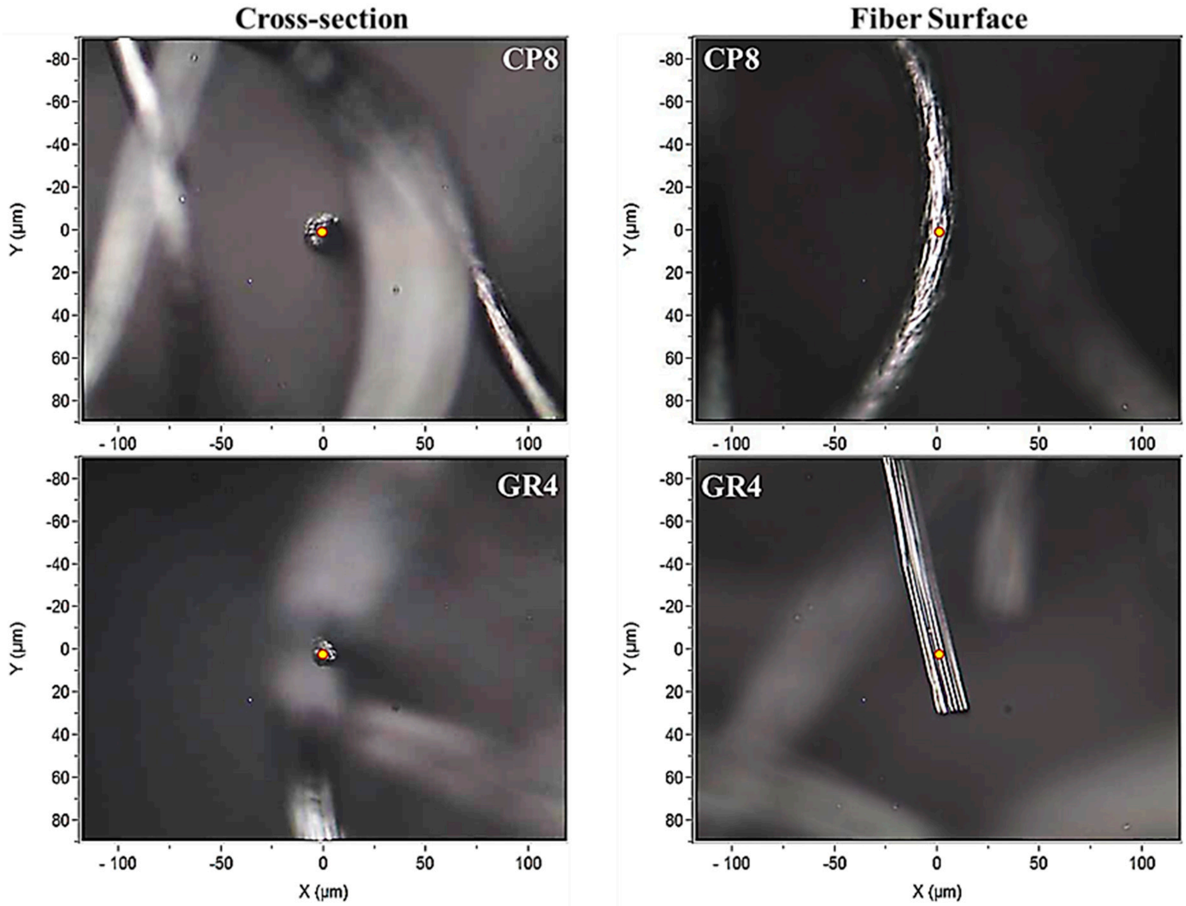

Figure S1. Raman laser spot position (yellow midpoint), for achieving Raman spectra for both cross-section (left) and surface (right micrographs) of ex-PAN (CP8: top row) and ex-Rayon (GR4: bottom row) carbon fibres.

materials

MDPI

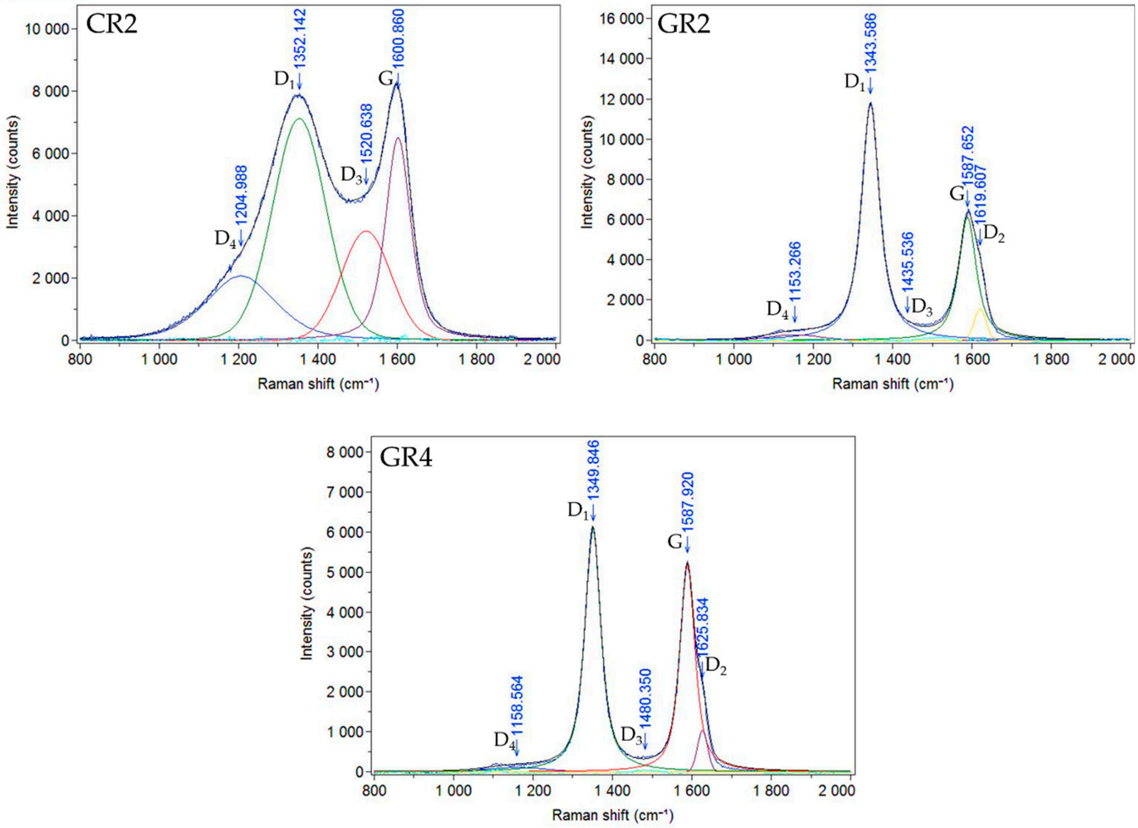

Figure S2. Deconvoluted Raman spectra of ex-Rayon CFs, where the scatter curves indicate the measured intensities and the solid smooth lines represent the fits.

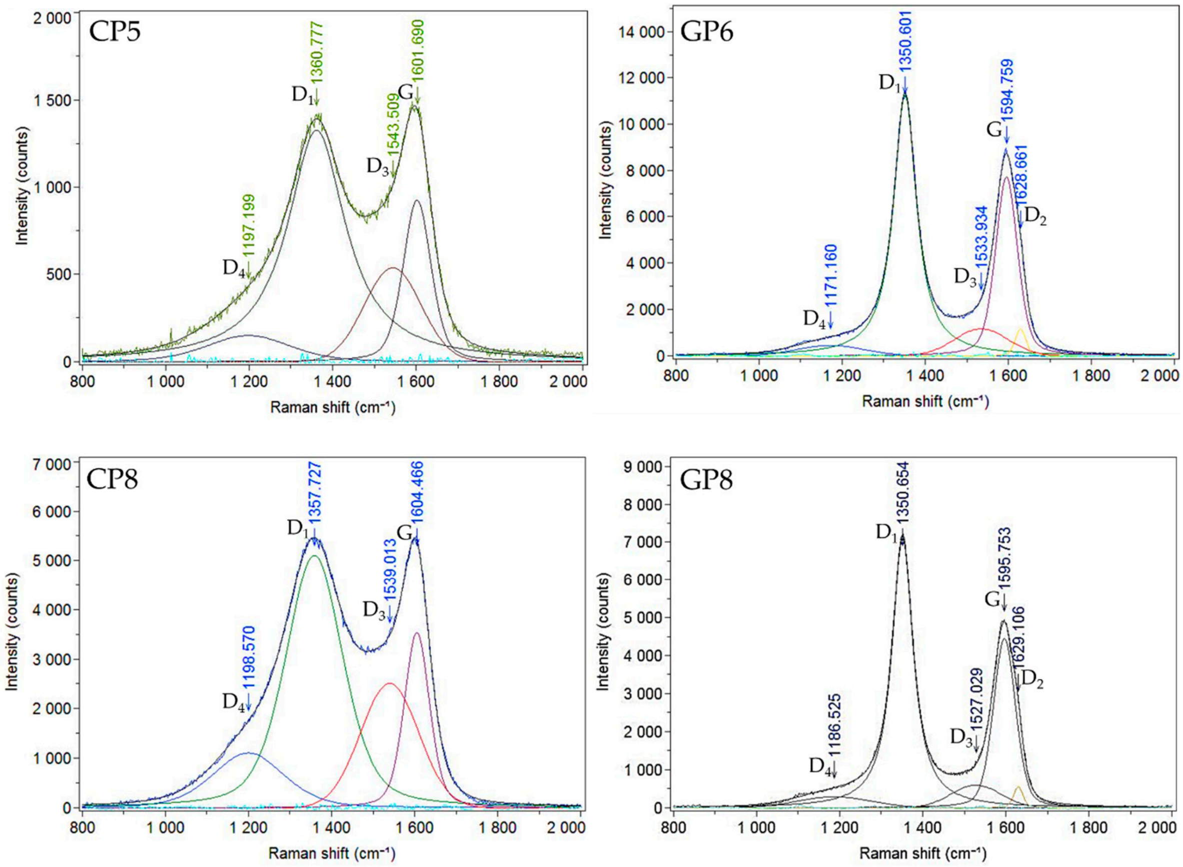

Figure S3. Same as Figure S2 but for ex-PAN CFs.

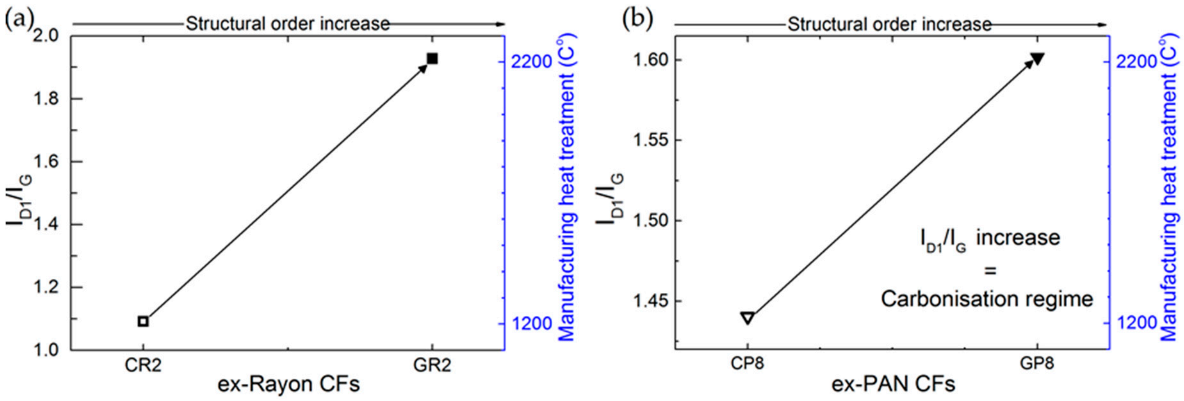

Figure S4. Intensity ratio between deconvoluted D_1 and G bands of corresponding: **(a)** ex-Rayon CFs and **(b)** ex-PAN CFs, with an arrow indicating the increase of structural order. Empty and full symbols stand for carbonised CFs and graphitised CFs, respectively.

Section 2. Textural Analysis

S2.1. N_2 and CO_2 Adsorption Isotherms of de-sized CFs before Chemical Reactivity Tests

De-sized CFs exhibited both type I and type II N_2 adsorption isotherms according to the IUPAC classification [1]. Thus, the adsorption isotherms of CR1, CR2, GR1, GR2, and GR3 are all of type I, suggesting microporous carbons (see Figures S5a,c). However, the smooth "knee" formation of the graphitised GR1 N_2 adsorption isotherms and higher adsorbed amounts indicate the existence of wider micropores and the appearance of mesopores, as compared to the other type I isotherms of microporous carbon fibres [2,3]. Graphitised GR3 and GR4 with improved structural order, on the contrary, exhibited N_2 isotherms (see Figure S5c), which have a sigmoidal shape with a sharp increase in the region of higher relative pressures, thus corresponding to the type II of IUPAC classification [4]. These adsorption results are in good agreement with the structural properties of the investigated CFs and the Franklin structural models of different carbon nanostructures and the presence of nanopores (see Figure 8).

CO_2 adsorption isotherms were performed in addition to those of N_2 (see Figures S5b,d)). They have a great advantage in studying microporous CFs and avoiding the well-known problems of restricted diffusion of gaseous molecules at low pressure [5]. The obtained CO_2 adsorption isotherms agree with the N_2 adsorption isotherms, so that the order of the CFs and the general differences in the amounts adsorbed are consistent in the two analyses. Therefore, using the 2D NLDFT-HS model, we combined the results of the CFs adsorption analysis, considered an effective characterisation tool for detailed and reliable textural analysis.

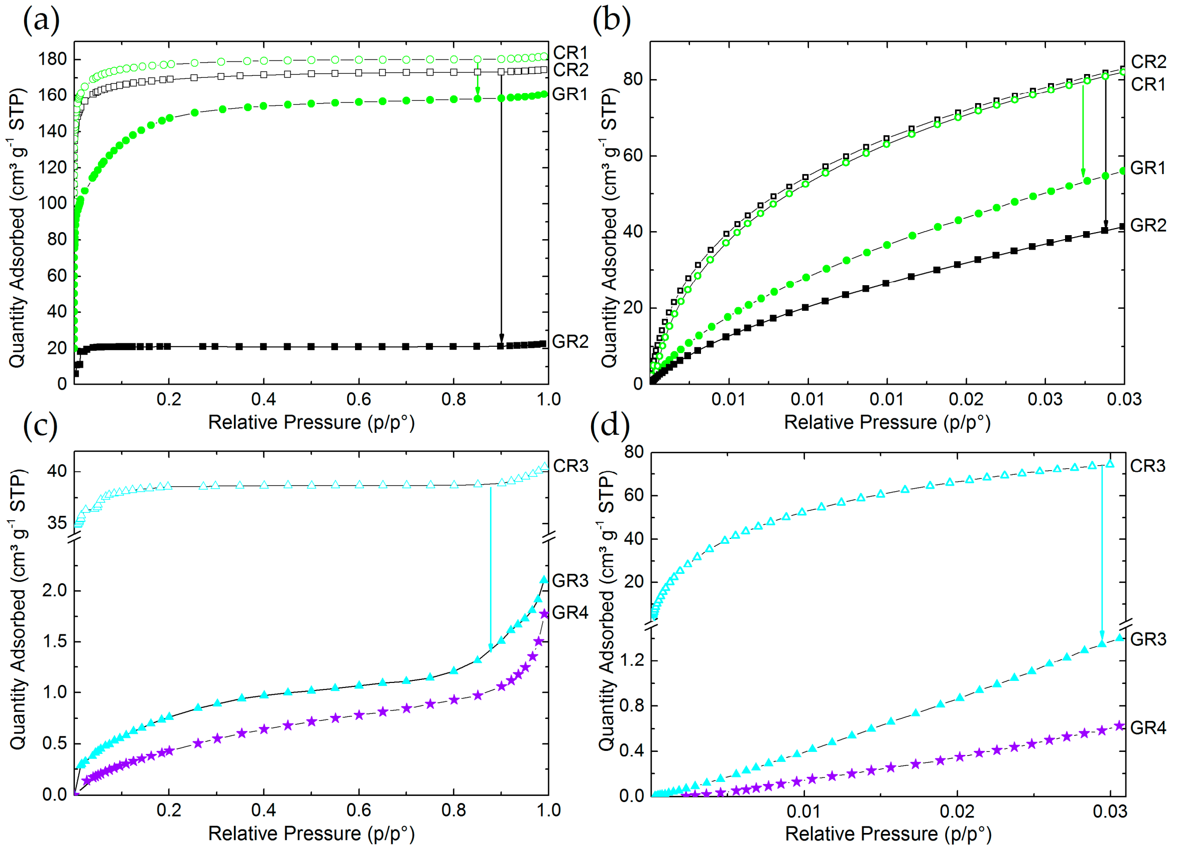

Figure S5. (a) and **(b)** N_2 adsorption isotherms at −196 °C, and **(c)** and **(d)** CO_2 adsorption isotherms at 0°C for de-sized CFs before reaction in molten Li-based salts.

S2.2. N_2 and CO_2 Adsorption Isotherms of CFs after Chemical Stability Tests in Molten LiOH and $Li_4(OH)_3Br$

Because of the low amount of material recovered after certain chemical reactivity tests, and because of the resultant microporous texture sometimes very narrow and poorly developed, it is difficult to achieve closed loop adsorption-desorption isotherms in some cases. As seen previously [6], for many CFs, adsorption equilibrium problems are observed due to the slow diffusion kinetics of N_2 in very narrow microporosity, at a temperature of analysis of −196 °C. Figures S6a,b initially compare the isotherms of CFs reacted in molten LiOH or $Li_4(OH)_3Br$ at the lowest reaction temperature (CR1, GR1, CR2, and GR2), where no hysteresis loop and narrower isotherm knees are observed. Hence, performing tests at a higher reaction temperature results in most cases in a change of the isotherm shape (to type I-IV) with the appearance of hysteresis loops (of type H2) and of steeper isotherm slope, attributable to the development of mesopores of ill-defined size and shape. Moreover, by performing the chemical reaction at 600 °C, a hysteresis loop and a wider isotherm knee are observed with LiOH but not with $Li_4(OH)_3Br$. An important slope of the isotherm, as seen for CR1a600 and CR2a600, indicates a widespread microporosity (based on adsorbed amounts up to a relative pressure of 0.2), followed by significant contribution of mesoporosity (also seen from the widest PSD shown in Figure 9a,b).

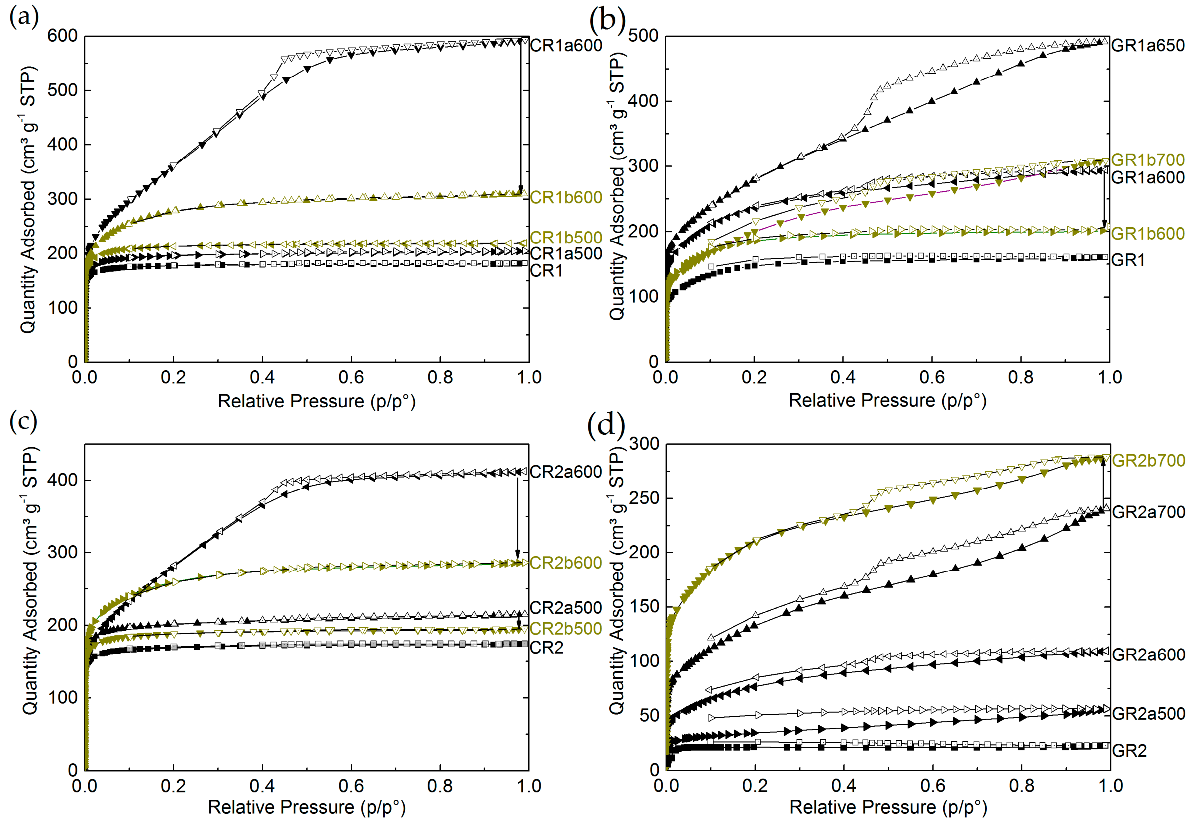

Figure S6. N_2 adsorption isotherms of corresponding carbonised and graphitised CFs before and after chemical reaction with LiOH (black symbols) and with $Li_4(OH)_3Br$ (green symbols). The arrow is shown for CFs reacted at a same temperature but in different molten salts.

The graphitised ex-Rayon CFs of improved purity and structural order show type II adsorption isotherms, typical of non-porous or macroporous absorbent with negligible hysteresis at relatively high pressure (see Figures S6c,d). After reaction in molten LiOH or $Li_4(OH)_3Br$ at the highest investigated temperature, the type of the isotherms remains unchanged, with only a change in the adsorbed amounts depending on the characteristics of the materials.

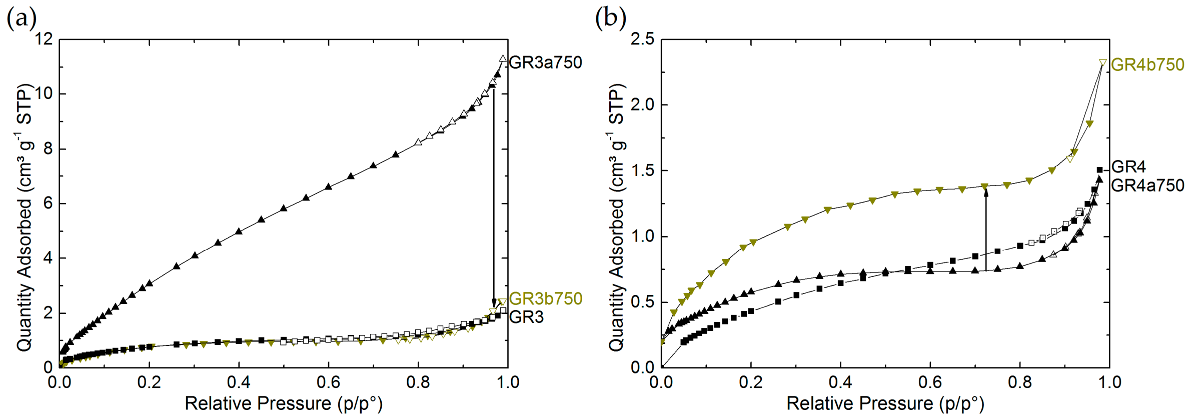

Figure S7. Same as Figure S6 but for graphitised ex-Rayon CFs (of highest structural order).

materials

MDPI

S2.3. Textural Properties of CFs before and after Chemical Reaction in Molten LiOH or $Li_4(OH)_3Br$

Table 1. Textural parameters from both N_2 and CO_2 isotherms calculated by the 2D NLDFT-HS model of ex-Rayon CFs examined before and after chemical reaction in molten LiOH (normal style) or $Li_4(OH)_3Br$ (underlined style). The bold style distinguishes graphitised CFs from carbonised CFs.

Carbon felt (CODE)	B-O[1] (%)	$S_{2D\ NLDFT\text{-}HS}$[2] ($m^2\ g^{-1}$)	$V_{\mu,\ 2D\ NLDFT\text{-}HS}$[3] ($cm^3\ g^{-1}$)	$V_{tot,\ 2D\ NLDFT\text{-}HS}$[4] ($cm^3\ g^{-1}$)	V_{meso}[5] ($cm^3\ g^{-1}$)	V_{meso} (%)
CR1	-	910.71	0.2775	0.2775	0.0000	0.00
CR1a500	5.6	930.99	0.3028	0.3047	0.0019	0.61
CR1a600	49.7	1057.69	0.3272	0.8564	0.5292	61.79
CR1b500	2.7	988.98	0.3281	0.3282	0.0001	0.04
CR1b600	20.4	996.27	0.3832	0.4474	0.0641	14.33
CR1b650	64.5	1011.14	0.2569	1.0438	0.7869	75.38
**GR1**	-	688.19	0.2652	0.2914	0.0262	8.99
**GR1a500**	0.9	787.86	0.3019	0.3800	0.0781	20.56
**GR1a600**	9.4	793.81	0.3047	0.4275	0.1229	28.74
**GR1a650**	38.7	869.19	0.2799	0.7058	0.4259	60.35
**GR1b600**	4.1	711.52	0.3985	0.4480	0.0495	12.10
**GR1b700**	33.8	847.35	0.2740	0.3118	0.0377	45.62
CR2	-	907.39	0.2732	0.2733	0.0001	0.04
CR2a500	5.2	985.71	0.3097	0.3217	0.0120	3.73
CR2a600	43.7	814.31	0.2747	0.5904	0.3158	53.48
CR2b500	2.9	941.69	0.2943	0.2950	0.0007	0.23
CR2b600	20.6	998.04	0.3627	0.4172	0.0545	13.06
CR2b650	72.6	1033.65	0.2667	1.1002	0.8335	75.76
**GR2**	-	424.11	0.1353	0.1355	0.0002	0.14
**GR2a500**	2.0	463.14	0.1523	0.1914	0.0390	20.40
**GR2a700**	85.9	622.23	0.1558	0.2288	0.0730	50.84
**GR2b500**	0.3	474.65	0.2085	0.4241	0.2156	3.62
**GR2b700**	20.8	656.69	0.1558	0.1617	0.0058	39.76
CR3	-	769.69	0.2029	0.2031	0.0002	0.10
CR3a500	1.7	754.85	0.2268	0.2268	0.0000	0.00
CR3a600	12.9	808.65	0.2835	0.2991	0.0156	5.22
**GR3**	-	10.92	0.0047	0.0071	0.0024	33.55
**GR3a600**	1.1	14.72	0.0063	0.0104	0.0041	39.36
**GR3a650**	3.4	17.28	0.0058	0.0140	0.0082	58.51
**GR3a700**	4.8	17.74	0.0033	0.0143	0.0110	76.72
**GR3a750**	28.8	43.24	0.0161	0.0323	0.0162	50.11
**GR3b750**	18.7	33.39	0.0136	0.0167	0.0032	18.91
**GR4**	-	6.62	0.0023	0.0045	0.0022	48.70
**GR4a600**	0.1	9.56	0.0040	0.0054	0.0013	25.19
**GR4a650**	3.4	11.68	0.0049	0.0075	0.0026	34.61
**GR4a700**	7.2	25.15	0.0100	0.0141	0.0042	29.53
**GR4a750**	18.4	13.85	0.0061	0.0077	0.0017	21.63
**GR4b750**	15.9	27.36	0.0114	0.0134	0.0020	14.95

1 Burn-off = 100 - percent of carbon disappeared per initial CF weight

2 Surface areas determined by 2D NLDFT-HS

3 Micropore volume (0.36 - 2 nm) determined by 2D NLDFT-HS

4 Total pore volume (0.36 - 10 nm) determined by 2D NLDFT-HS

5 Mesopore volume (2-10 nm) = $V_{tot,\ 2D\ NLDFT\text{-}HS} - V_{\mu,\ 2D\ NLDFT\text{-}HS}$

materials MDPI

Table 2. Same as Table S1 but for ex-PAN CFs.

Carbon felt (CODE)	B-O[1] (%)	$S_{2D\ NLDFT\text{-}HS}$[2] ($m^2\ g^{-1}$)	$V_{\mu,\ 2D\ NLDFT\text{-}HS}$[3] ($cm^3\ g^{-1}$)	$V_{tot,\ 2D\ NLDFT\text{-}HS}$[4] ($cm^3\ g^{-1}$)	V_{meso}[5] ($cm^3\ g^{-1}$)	V_{meso} (%)
CP5	-	-	-	-	-	-
CP5a500	0.9	15.29	0.0061	0.0082	0.0021	25.08
CP5a750	31.7	18.04	0.0073	0.0103	0.0030	28.90
**GP6**	-	-	-	-	-	-
**GP6a750**	15.5	24.54	0.0104	0.0131	0.0027	20.84
CP8	-	-	-	-	-	-
CP8a650	14.7	33.75	0.0100	0.0257	0.0157	61.14
CP8a700	49.0	29.30	0.0104	0.0194	0.0091	46.68
**GP8**	-	-	-	-	-	-
**GP8a650**	1.2	10.29	0.0041	0.0046	0.0005	10.80
**GP8a750**	10.0	13.65	0.0057	0.0067	0.0010	14.58

1 Burn-off = 100 - precent of carbon disappeared per initial CF weight
2 Surface areas determined by 2D NLDFT-HS
3 Micropore volume (0.36 – 2 nm) determined by 2D NLDFT-HS
4 Total pore volume (0.36 – 10 nm) determined by 2D NLDFT-HS
5 Mesopore volume (2–10 nm) = $V_{tot,\ 2D\ NLDFT\text{-}HS}$ - $V_{\mu,\ 2D\ NLDFT\text{-}HS}$

Section 3. Scanning Electron Microscopy

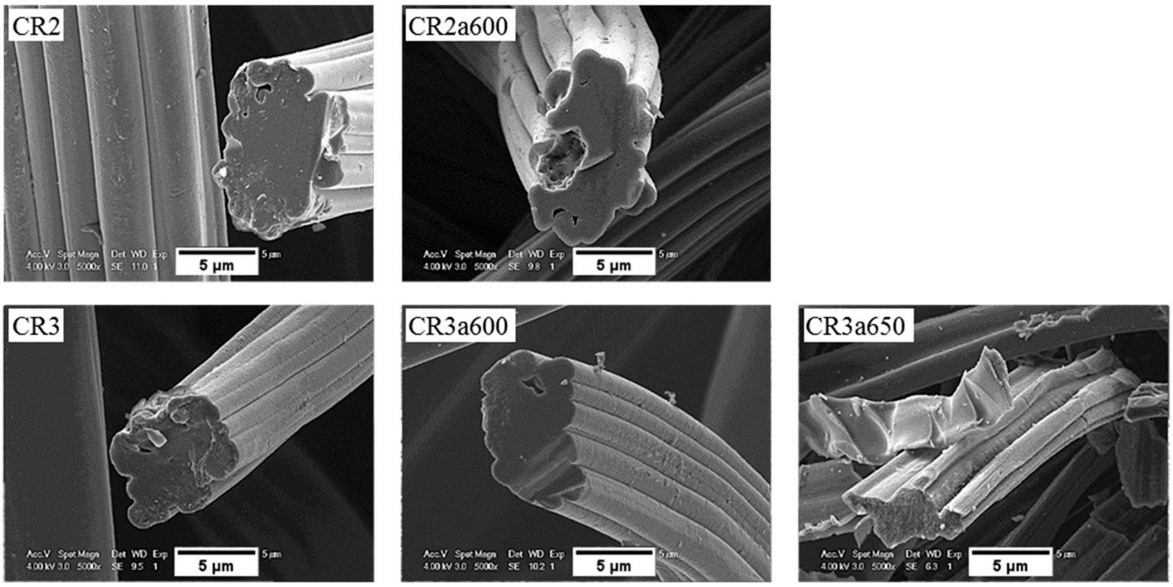

Figure S8. SEM micrographs of de-sized ex-Rayon CFs before (left) and after (middle and right) reaction with LiOH at different temperatures.

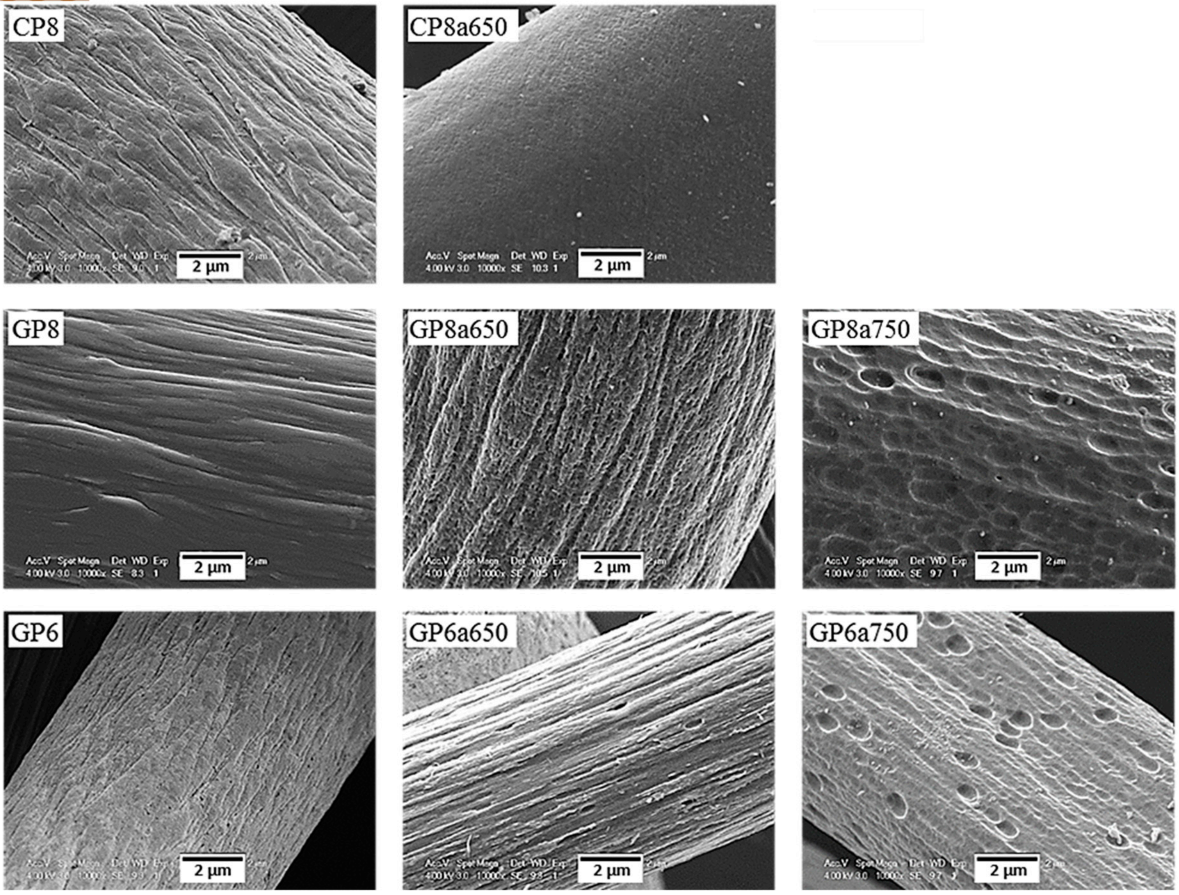

Figure S9. Same as Figure S8 but for de-sized ex-PAN CFs.

Section 4. Estimation of CFs Chemical Stability at the TES Application Temperature

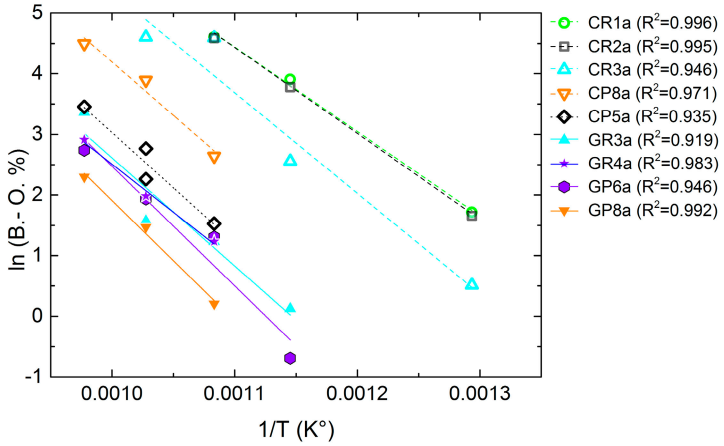

Figure S10. Application of equation (4) to the experimentally derived B-O data of most CFs shown in Figures 7(a) and (b). Carbonised or graphitised CFs are represented by empty or solid symbols and dashed or solid linear fits, respectively.

References

1. Sing, K.S.W. Physisorption of gases by carbon blacks. *Carbon* **1994**, *32*, 1311–1317 doi: 10.1016/0008-6223(94)90117-1.
2. Lu, A.-H.; Zheng, J.-T. Study of Microstructure of High-Surface-Area Polyacrylonitrile Activated Carbon Fibers. *J. Colloid Interface Sci.* **2001**, *236*, 369–374 doi: 10.1006/jcis.2000.7425.

3. Maciá-Agulló, J.A.; Moore, B.C.; Cazorla-Amorós, D.; Linares-Solano, A. Activation of coal tar pitch carbon fibres: Physical activation vs. chemical activation. *Carbon* **2004**, *42*, 1367–1370 doi: 10.1016/j.carbon.2004.01.013.
4. Jagannathan, S.; Chae, H.G.; Jain, R.; Kumar, S. Structure and electrochemical properties of activated polyacrylonitrile based carbon fibers containing carbon nanotubes. *J. Power Sources* **2008**, *185*, 676–684 doi: 10.1016/j.jpowsour.2008.08.093.
5. Landers, J.; Gor, G.Y.; Neimark, A.V. Density functional theory methods for characterization of porous materials. *Colloids Surf. Physicochem. Eng. Asp.* **2013**, *437*, 3–32 doi: 10.1016/j.colsurfa.2013.01.007.
6. Huidobro, A.; Pastor, A.C.; Rodrı́guez-Reinoso, F. Preparation of activated carbon cloth from viscous rayon: Part IV. Chemical activation. *Carbon* **2001**, *39*, 389–398 doi: 10.1016/S0008-6223(00)00131-7.

© 2019 by the authors. Submitted for possible open access publication under the terms and conditions of the Creative Commons Attribution (CC BY) license (http://creativecommons.org/licenses/by/4.0/).
